# Structural basis of ligand interaction with atypical chemokine receptor 3

**DOI:** 10.1038/ncomms14135

**Published:** 2017-01-18

**Authors:** Martin Gustavsson, Liwen Wang, Noortje van Gils, Bryan S. Stephens, Penglie Zhang, Thomas J. Schall, Sichun Yang, Ruben Abagyan, Mark R. Chance, Irina Kufareva, Tracy M. Handel

**Affiliations:** 1 Skaggs School of Pharmacy and Pharmaceutical Sciences, University of California, San Diego, 9500 Gilman Drive, MC 0684, La Jolla, California, 92093, USA; 2 Center for Proteomics and Bioinformatics and Department of Nutrition, Case Western Reserve University School of Medicine, 10009 Euclid Avenue, Cleveland, Ohio 44109, USA; 3 ChemoCentryx Inc., 850 W Maude Avenue, Mountain View, California 94043, USA

## Abstract

Chemokines drive cell migration through their interactions with seven-transmembrane (7TM) chemokine receptors on cell surfaces. The atypical chemokine receptor 3 (ACKR3) binds chemokines CXCL11 and CXCL12 and signals exclusively through β-arrestin-mediated pathways, without activating canonical G-protein signalling. This receptor is upregulated in numerous cancers making it a potential drug target. Here we collected over 100 distinct structural probes from radiolytic footprinting, disulfide trapping, and mutagenesis to map the structures of ACKR3:CXCL12 and ACKR3:small-molecule complexes, including dynamic regions that proved unresolvable by X-ray crystallography in homologous receptors. The data are integrated with molecular modelling to produce complete and cohesive experimentally driven models that confirm and expand on the existing knowledge of the architecture of receptor:chemokine and receptor:small-molecule complexes. Additionally, we detected and characterized ligand-induced conformational changes in the transmembrane and intracellular regions of ACKR3 that elucidate fundamental structural elements of agonism in this atypical receptor.

## Abstract (web summary)

The atypical chemokine receptor 3 (ACKR3) is important for cell migration in development and cancer. Here the authors combine radiolytic footprinting, disulfide trapping, mutagenesis and molecular modelling to characterize the ligand interactions and ligand-induced conformational changes in ACKR3.

Positional control of cell movement plays a crucial role in development, the innate and adaptive arms of the immune system and regulation of a number of other physiological functions such as angiogenesis and wound repair^1^. Guidance cues are provided by small globular chemoattractant proteins called chemokines that accumulate in gradients on cell surfaces and the extracellular matrix and are interpreted as directional signals by chemokine receptors on migrating cells. Most chemokine receptors are seven-transmembrane G-protein-coupled receptors (GPCRs) that activate Gαi-dependent intracellular pathways in response to chemokine binding. However, a few chemokine receptors signal via other mechanisms and are therefore referred to as atypical chemokine receptors (ACKRs)^2^.

ACKR3 (also known as CXCR7) is an atypical receptor that binds chemokines CXCL11 (a.k.a. ITAC, shared with CXCR3) and CXCL12 (a.k.a. SDF-1, shared with CXCR4)^3^. It does not couple to G proteins but signals through alternate pathways including β-arrestins^4^. ACKR3 expression is upregulated in several cancers and the associated tumor vasculature^5^ where it cooperates with CXCR4, a receptor heavily implicated in cancer growth and metastasis^6^. Although there have been contradictory reports where ACKR3 enhances CXCR4-mediated metastasis and others where it inhibits CXCR4 (refs 5, 7), emerging evidence suggests that it can signal through β-arrestin to ERK1/2, AKT and other pathways to promote tumor migration and survival as well as the survival and self-renewal of cancer stem cells^8^. In addition to its unusual signalling properties, ACKR3 also acts as a scavenger of extracellular CXCL12 to establish chemokine levels that maintain cellular responsiveness by preventing excessive desensitization and downregulation of CXCR4. In this context, ACKR3-expressing cells in a primary breast tumor enhanced the metastasis of CXCR4-expressing breast cancer cells^9^. Similarly, scavenging of CXCL12 by ACKR3 has been shown to maintain CXCR4 responsiveness in migrating cortical interneurons^10^ and the lateral line primordium of zebrafish^11^. By contrast, pharmacological inhibition of ACKR3 has been shown to cause pronounced increases in plasma CXCL12 levels^9^ with associated impairment of leukocyte migration towards CXCL12, likely because of CXCR4 downregulation. These and other studies suggest that ACKR3 may be a good therapeutic target for cancer^12^. In support of this concept, short interfering RNA, small molecules and nanobodies against ACKR3 have been shown to slow cancer progression through effects on proliferation, survival signalling, metastasis and angiogenesis^12^,^13^.

Crystal structures have been determined for chemokine receptors CXCR4 and CCR5 in complex with small molecules and, more recently, for CXCR4 in complex with the viral chemokine vMIP-II and for US28 in complex with human CX3CL1 (refs 14, 15, 16, 17). However, despite its promise as a therapeutic target, there is currently minimal structural data for ACKR3. This is not surprising as the structural biology of seven transmembrane (7TM) receptors remains challenging due to their inherent flexibility and low stability, their limited surface area for forming crystal contacts, the need for slow off-rate ligands for crystallization and many other technical hurdles^18^. Here, we produce stable complexes between ACKR3 and CXCL12 as well as the small-molecule partial agonist CCX777. Radiolytic footprinting, disulfide trapping and mutagenesis approaches are combined to map the ligand interaction interfaces and the effects of ligand binding on the structure of ACKR3. Using a total of ∼100 probes located throughout the ACKR3 and CXCL12 sequences, we identify interaction sites between ACKR3 and these ligands as well as conformational changes in the transmembrane helical domain of ACKR3 that are linked to activation of the receptor. Finally, we utilize experimentally guided homology modelling to produce models of ACKR3:CXCL12 and ACKR3:CCX777 complexes that reveal insights into receptor:ligand recognition and may guide drug discovery efforts.

## Results

### Characterization of ACKR3 ligand pharmacology

**Figure 1:**
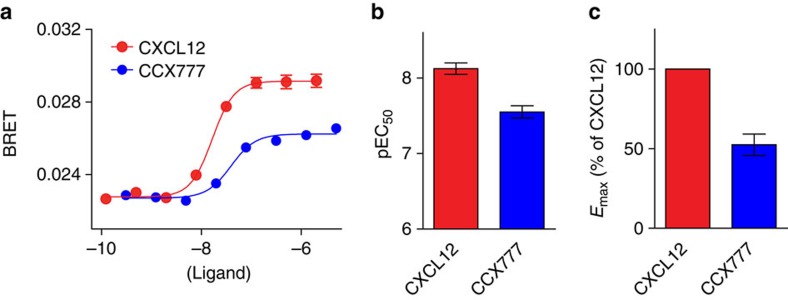
Functional characterization of CXCL12 and CCX777. (**a**) Dose–response curves of β-arrestin-2-GFP10 recruitment to ACKR3-Rluc3 in HEK293 cells. Curves are representative of 10 independent experiments and error bars represent standard errors of the mean of three replicates. (**b**, **c**) pEC_50_ and *E*_max_ values determined as the average and standard error of the mean of 10 independent experiments. *E*_max_ values were determined as the difference between the start and end values of dose–response curves and were normalized to CXCL12. CCX777 has lower *E*_max_ and pEC_50_ than CXCL12 as determined from two-sided, unpaired *t*-test with *P*=0.002 and *P*<0.0001, respectively.

To probe the structure and ligand interactions of ACKR3, we characterized ACKR3 in complex with two different ligands, the chemokine CXCL12 and the small-molecule CCX777. CXCL12 is a known agonist of β-arrestin signalling^4^. By contrast, the pharmacological response of ACKR3 to CCX777 has not been characterized, although similar synthetic ligands are known to induce β-arrestin recruitment^19^. To compare the functional responses of ACKR3 to CCX777 and CXCL12, we utilized a β-arrestin recruitment assay based on bioluminescence resonance energy transfer (BRET). ACKR3 fused to *Renilla* luciferase 3 (ACKR3-Rluc3) was transiently transfected into HEK293 cells stably expressing β-arrestin-2 fused to green fluorescent protein 10 (GFP10) and recruitment of β-arrestin-2 was measured as an increase in BRET after stimulation with ligand. Dose–response curves show that CCX777 acts as a partial agonist of β-arrestin-2 recruitment to ACKR3 with an efficacy of 52±7% and a fourfold lower potency (33±6 versus 8.5±1.3 nM) than CXCL12 (Fig. 1). From a functional perspective ACKR3:CXCL12 therefore represents a complex that is biased towards a more active receptor conformation than the partially active ACKR3:CCX777 complex, allowing comparison of the differences between the two conformations by radiolytic footprinting (described below).

### Production of stable ACKR3 complexes with ligands

**Figure 2:**
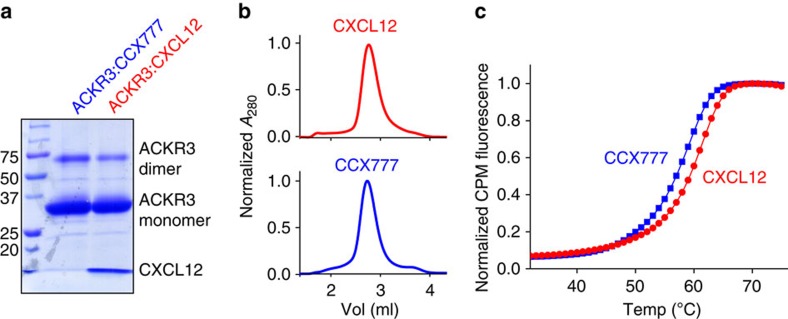
Characterization of ACKR3 complexes in DDM/CHS micelles. (**a**) A strong band corresponding to monomeric ACKR3 and a minor band corresponding to dimeric receptor can be seen on a 10% SDS–PAGE gel. A lower molecular weight band corresponding to CXCL12 is seen only for the ACKR3:CXCL12 complex. A full, uncropped version of the gel is shown in Supplementary Fig. 7. (**b**) SEC trace of ACKR3 in complex with CXCL12 and CCX777. The sharp peak eluting at ∼2.8 ml shows that both samples are monodisperse in DDM/CHS micelles. (**c**) Thermal unfolding of ACKR3 complexes using CPM fluorescence^21^. *T*_m_ values were 59 and 61 °C for ACKR3:CCX777 and ACKR3:CXCL12 complexes, respectively.

To produce an ACKR3:CXCL12 complex, receptor and chemokine were coexpressed in *Spodoptera frugiperda* (Sf9) cells and the complex was purified in *n*-dodecyl β-D-maltoside/cholesteryl hemisuccinate (DDM/CHS) using affinity chromatography (Fig. 2a and Supplementary Fig. 7)^20^. To form a complex between ACKR3 and the small-molecule ligand CCX777, Sf9 membranes expressing ACKR3 were incubated with a large excess of CCX777 before purification (Fig. 2a). Similar to other 7TM receptors, ACKR3 is unstable in the absence of ligand, which makes experiments with apo receptor impossible (Supplementary Fig. 1). In contrast, ACKR3:CXCL12 and ACKR3:CCX777 complexes are monodisperse and stable with unfolding midpoints of ∼60 °C as determined by analytical size-exclusion chromatography (SEC; Fig. 2b) and thermal unfolding experiments^21^ (Fig. 2c), respectively. These complexes are suitable for mapping binding interfaces and conformational changes by radiolytic oxidation.

### Binding interfaces mapped by radiolytic footprinting

Radiolytic protein footprinting is a structural mass spectrometry (MS) technique where synchrotron radiation is used to generate hydroxyl radicals that covalently modify side chains proximal to either bulk solvent or structural water molecules^22^. The resulting mass shifts related to specific peptide side-chain oxidations can be detected using protease digestion in combination with tandem MS. As the rate of radiolytic oxidation of a side chain is proportional to its solvent exposure, the technique can be used to map interaction interfaces as sets of residues that are buried upon complex formation. The method can also report on conformational transitions that change the exposure of individual residues to bulk solvent or their interaction with structural waters^23^.

In this work, we measured residue oxidation rates of three samples: CXCL12 alone, ACKR3:CXCL12 and ACKR3:CCX777. Proteolytic digestion of oxidized CXCL12 samples yielded 11 peptides that covered 87% of the sequence (Supplementary Fig. 2a). For ACKR3:CCX777 and ACKR3:CXCL12 complexes, digestion with pepsin yielded 337 unique peptides covering 100% of ACKR3 (Supplementary Fig. 2b), while digestion with a combination of trypsin and AspN gave 28 peptides and 51% sequence coverage (Supplementary Fig. 2c). From these peptides, oxidation rates were determined, providing discrete readouts for 62 and 25 unique sites on ACKR3 and CXCL12, respectively (see Supplementary Fig. 3 for representative dose–response plots and Supplementary Tables 1 and 2 for oxidation rates of specific residues); these data represent a total sequence coverage and structural mapping by MS that by far exceeds what has been previously achieved for any 7TM receptor complex^23^,^24^,^25^,^26^.

**Figure 3:**
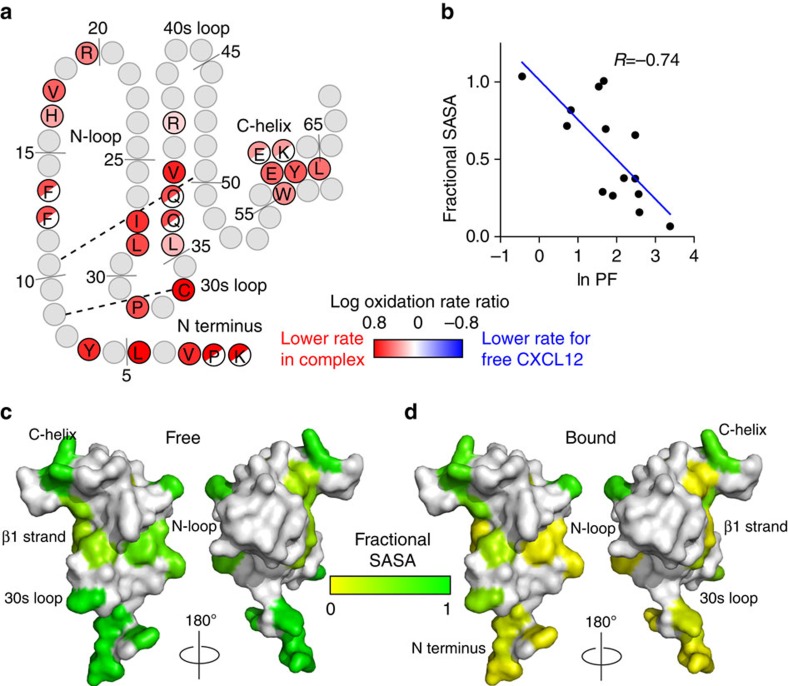
Radiolytic footprinting of CXCL12. (**a**) Radiolytic footprinting results mapped onto a topology plot of CXCL12. One letter amino-acid codes are indicated for all residues with detectable oxidation rates. CXCL12 residues with reduced oxidation rates in the ACKR3:CXCL12 complex are coloured red, residues where data are missing are coloured grey and half-coloured circles signify that an oxidation rate was mapped to one or both of two neighbouring residues. (**b**) Correlation between the natural log of the PF calculated for all +16 Da oxidized residues and the fractional side-chain SASA (0 corresponds to no solvent exposure and 1 corresponds to complete exposure) calculated from the crystal structure of CXCL12 (PDB: 1a15)^28^. (**c**) Fractional SASA for free CXCL12 mapped onto the crystal structure and coloured according to SASA. (**d**) Predicted SASA values for CXCL12 in complex with ACKR3 mapped onto the crystal structure of CXCL12 (PDB: 1a15)^28^.

Nearly all of the measured oxidation rates for CXCL12 are decreased on binding to ACKR3 (Fig. 3a and Supplementary Table 1). To interpret this observation in a structural context, we converted the oxidation rates for side chains exhibiting +16 Da oxidations in free CXCL12 into protection factors (PFs) using residue-specific coefficients of oxidation propensity^27^ (see Supplementary Table 3). The natural logarithms of the measured PFs inversely correlate (*R*=–0.74) with the solvent-accessible surface areas (SASAs) calculated from the CXCL12 X-ray structure^28^ (Fig. 3b), confirming that the oxidation rates reliably report on residue bulk solvent exposure. Using this linear correlation and ln PF values for ACKR3-bound CXCL12, we then predicted the SASA values (and fractional SASA (fSASA) values) for CXCL12 in the context of the ACKR3:CXCL12 complex^27^,^29^. This analysis shows that when in complex with the receptor, most chemokine residue SASA values (of those calculated) are significantly lower than for CXCL12 alone (Fig. 3c,d), with the largest changes concentrated in the chemokine N terminus (where most probe residues become entirely buried). Other significant changes involve residues F13/F14, H17, V18, R20 (the so-called N-loop), residues P32 and C34 in the 30s loop, E60, Y61 and L62 in the C-helix, Q37/Q38 and V39 in the β_2_ strand and residues I28 and L29 in the β_1_ strand (that facilitates dimerization of CXCL12 when it is alone in solution^30^). These data reveal an extensive receptor:chemokine interaction interface, with multiple receptor elements essentially wrapping around the chemokine.

**Figure 4:**
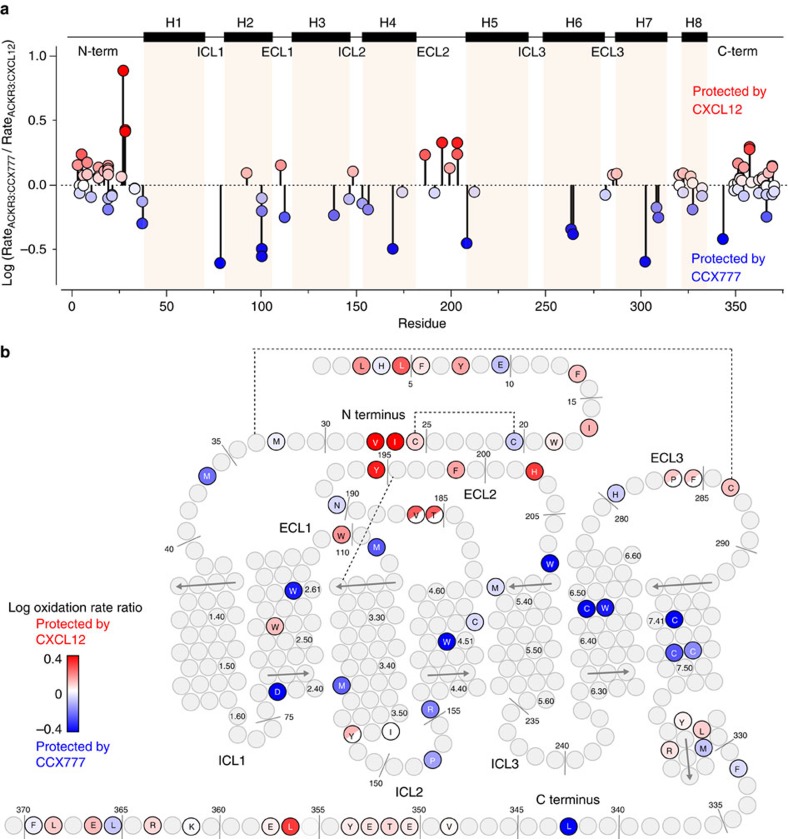
Radiolytic footprinting of ACKR3 complexes. (**a**) All detectable ACKR3 oxidation rates mapped onto the sequence. Rates are shown as the log of Rate_ACKR3:CCX777_/Rate_ACKR3:CXCL12_. Residues are coloured using a gradient where blue corresponds to reduced oxidation rates in the ACKR3:CCX777 complex (protection by CCX777) and red corresponds to reduced oxidation rates in the ACKR3:CXCL12 complex. (**b**) Radiolytic footprinting results mapped onto a topology plot of ACKR3. Residues for which oxidation rates were measured are highlighted and coloured according to the scheme in (**a**). Residues where multiple rates were measured are coloured by the average log (Rate_ACKR3:CCX777_/Rate_ACKR3:CXCL12_ ) of the multiple oxidation events. Half-coloured circles signify that an oxidation rate was mapped to one or both of two neighbouring residues.

Although we can quantitatively interpret oxidation rates for the soluble chemokine, such an analysis cannot be carried out for a receptor for which no definitive structural information is available and for which many of the oxidation sites are buried in the transmembrane region. Thus, we performed a relative comparison by calculating the ratios of rates for all detected species between the chemokine and the small-molecule bound states of ACKR3. As demonstrated by Fig. 4 (see also Supplementary Fig. 4 and Supplementary Table 2), a number of N-terminal residues of the receptor (including L3, L5, I27 and V28), as well as residues in ECL2 (Y195, V186 and H203) are protected by CXCL12. These findings are consistent with previous mutagenesis studies showing that mutations in ECL2 of ACKR3 lead to reduced potency of CXCL12-induced β-arrestin-2 recruitment^31^. In comparison with the protection of CXCL12, the reductions of oxidation rates in the receptor N terminus upon binding the chemokine are modest. This can likely be explained by the N terminus being partially folded and therefore protected from solvent even in the ACKR3:CCX777 sample. Corroborating this hypothesis, the oxidation rates of the ACKR3 N terminus are lower than what would be expected for an unfolded peptide^27^ (Supplementary Table 2). In contrast to the greater protection of residues in the extracellular domains by CXCL12 than CCX777, multiple residues in the transmembrane region are more protected in the CCX777 complex than in the CXCL12 complex.

Binding of chemokines to receptors has been shown to involve two main interaction sites referred to as chemokine recognition sites (CRS) 1 and 2 (ref. 14). CRS1 includes interactions between the N terminus of the receptor and the globular core of the chemokine, while CRS2 interactions involve the N-terminal ‘signalling domain' of the chemokine and the transmembrane (orthosteric) binding pocket of the receptor. As a small-molecule ligand, CCX777 is expected to occupy the orthosteric ligand-binding pocket of ACKR3, and overlap the chemokine N terminus in the CRS2 region. Thus, in the absence of an apo receptor sample, ACKR3:CCX777 mimics a ‘chemokine-free' receptor in the CRS1 region (which enables characterization of CRS1 interactions between CXCL12 and ACKR3), while simultaneously allowing for comparison of small molecule and chemokine binding in CRS2. The observed oxidation rate changes are in perfect agreement with this model. Additionally, the changes in oxidation rates of numerous TM domain and intracellular residues upon full agonist (chemokine) binding as compared to partial agonist (small-molecule) binding is expected to report on the conformational differences that the receptor undergoes on activation.

### Pairwise residue proximities probed by cysteine trapping

Radiolytic footprinting identifies individual residues that are part of interaction interfaces, but it does not report on pairwise interaction geometry. To obtain such pairwise information, we probed interactions between ACKR3 and CXCL12 using disulfide trapping, as previously described for other receptor:chemokine complexes^14^,^32^,^33^. In this technique, strategically designed Cys mutations are individually introduced into the receptor and chemokine of interest. If the Cys residues are proximal and adopt a favourable geometry in the complex, they spontaneously form a disulfide bond, and the resulting irreversible complex can be identified using SDS–polyacrylamide gel electrophoresis (SDS–PAGE) and western blots of purified samples; moreover, the ratio of complexed to uncomplexed receptor provides an indication of the efficiency of the crosslink.

**Figure 5:**
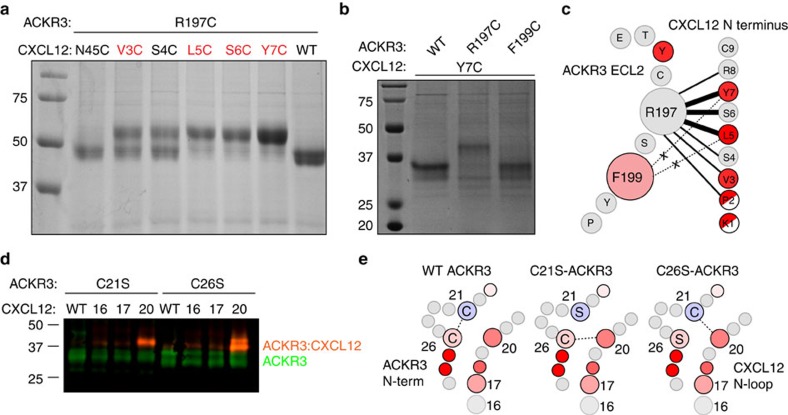
Cysteine trapping of the complex between ACKR3 and CXCL12. (**a**) CRS2 interactions detected using SDS–PAGE of purified ACKR3:CXCL12 complexes in the absence of reducing agent. Upper bands correspond to the crosslinked complex and lower bands to free receptor. Crosslinking of thermostabilized apocytochrome b562 (bRIL)-R197C-ACKR3 with Cys mutants in the N terminus of CXCL12. No crosslinking is seen for control samples N45C-CXCL12 or WT-CXCL12. (**b**) Crosslinking of Y7C-CXCL12 with ECL2 mutants of ACKR3. R197C-ACKR3 efficiently crosslinks with the chemokine, while no crosslinking is detected for F199C-ACKR3 or WT-ACKR3. (**c**) Schematic summary of disulfide trapping experiments involving ECL2 of ACKR3 and the N terminus of CXCL12. Residues are coloured based on radiolytic footprinting results (see Figs 3 and 4). Crosslinked sites are connected with solid lines, where the line thickness is proportional to crosslinking efficiency. Dashed lines indicate unsuccessful cross-links. (**d**) Cysteine trapping of CRS1 interactions detected by western blot of ACKR3:CXCL12 complexes under non-reducing conditions. ACKR3-FLAG and CXCL12-HA were detected with primary anti-FLAG and anti-HA antibodies and fluorescent secondary antibodies; 16, 17 and 20 refer to the residue in CXCL12 mutated to Cys. (**e**) Schematic representation of CRS1 disulfide trapping experiments. Mutation of C21 or C26 to Ser gives a free Cys side chain that can be crosslinked to residue 20 of CXCL12. Residues are coloured based on radiolytic footprinting results (see Figs 3 and 4). Full, uncropped versions of the gels and blots are shown in Supplementary Fig. 7.

Assuming that the general concept of CRS1/2 receptor:chemokine interactions applies to the ACKR3:CXCL12 complex, we hypothesized that protection of ECL2 of ACKR3 was partly due to CRS2 interactions with the N terminus of CXCL12. Figure 5a–c shows that a specific cysteine mutation (R197C) in ECL2 of ACKR3 crosslinks to residues in the N terminus of CXCL12, and that the most efficient crosslink is formed with Y7C of CXCL12. While some crosslinking may be nonspecific and not representative of the final bound states, this mutant produced almost 100% disulfide-trapped complex when coexpressed with R197C-ACKR3, suggesting high compatibility with the preferred interaction geometry. Thermal unfolding experiments show that of all crosslinked complexes, R197C-ACKR3:Y7C-CXCL12 is the most stable complex. Its stability exceeds that of non-covalent R197-ACKR3:CXCL12 by ∼20 °C, which indicates that it is the best mimic of the native ACKR3:CXCL12 geometry (Supplementary Fig. 5). Although in radiolytic footprinting experiments, no oxidation rates could be measured directly for R197 (Arg does not oxidize efficiently^27^), the observed strong protection of the neighbouring Y195 and partial protection of F199 are consistent with this pairwise interaction; similarly, earlier mutagenesis experiments also support the role of R197 (ref. 31). Importantly, contacts between Y7 of CXCL12 and ECL2 of ACKR3 would orient the preceding residues of the CXCL12 N terminus into the orthosteric pocket of ACKR3 to trigger signalling, consistent with expectations from the CRS1/2 ‘two-site model'.

Protection of the ACKR3 N terminus and the CXCL12 N-loop is likely due to CRS1 interactions with the body of the chemokine^14^. However, the N terminus of ACKR3 contains two native Cys residues at positions 21 and 26, which makes introduction of additional Cys residues challenging because of increased probability of disulfide shuffling and protein misfolding. Due to their position in the extracellular region, we hypothesized that C21 and C26 form an intramolecular disulfide bridge in ACKR3. Therefore, to probe intermolecular CRS1 proximities in the ACKR3:CXCL12 complex, we used an alternative approach where the two Cys residues were separately mutated to Ser leaving a single native Cys residue intact. Both mutants (C21S-ACKR3 and C26S-ACKR3) crosslink efficiently and specifically to R20C-CXCL12 (Fig. 5d,e), confirming their proximity to the junction between the N-loop and β_1_ strand of the chemokine. Supporting the C21–C26 disulfide hypothesis, R20C-CXCL12 does not crosslink with the wild-type (WT) receptor. Interactions in this region likely explain the observed radiolytic protection of receptor residues I27 and V28 when in complex with CXCL12.

Chemokine receptors have two conserved disulfides, one linking the N terminus with ECL3 and the other connecting ECL2 with the top of TM3. However, thus far, no other chemokine receptor has been reported to have a third disulfide in its N terminus. To further investigate the possibility of the unique intramolecular disulfide between C21 and C26 of ACKR3, we performed protease digestion followed by MS/MS experiments under non-reducing conditions, with free cysteines blocked by iodoacetamide (IA)^34^. Using pepsin and/or trypsin/AspN digests, several unique peptides containing disulfide bond C21–26 are detected with high confidence (Supplementary Table 4), confirming the presence of the third disulfide bond.

### Probing complex geometry by site-directed mutagenesis

Based on the disulfide-trapping experiments and previous receptor structures, CCX777 and the N terminus of CXCL12 are both predicted to regulate ACKR3 signalling through interactions with the orthosteric pocket of ACKR3. To probe this region and to complement our radiolytic footprinting and disulfide-trap mapping of ACKR3:ligand interaction determinants, we introduced single Ala mutations and analysed their effects on the potency and efficacy of ACKR3 signalling in response to both CXCL12 and CCX777 using the BRET-based β-arrestin-2 recruitment assay. A total of 12 Ala mutations were introduced into the ACKR3-Rluc3 construct and expressed in HEK293 cells stably expressing GFP10-β-arrestin-2. For all mutants, total expression levels are similar to WT ACKR3 (Supplementary Fig. 6b). Surface expression levels correlate well with total expression for all mutants except F124^3.32^A, which shows reduced surface expression (∼60%; Supplementary Fig. 6a) with WT-like total expression. For this mutant, the reduced surface expression must be taken into account in the course of interpretation of the BRET β-arrestin-2 recruitment experiments. For all other mutants, BRET interpretation is relatively straightforward as detailed in the legend of Supplementary Fig. 6.

**Figure 6:**
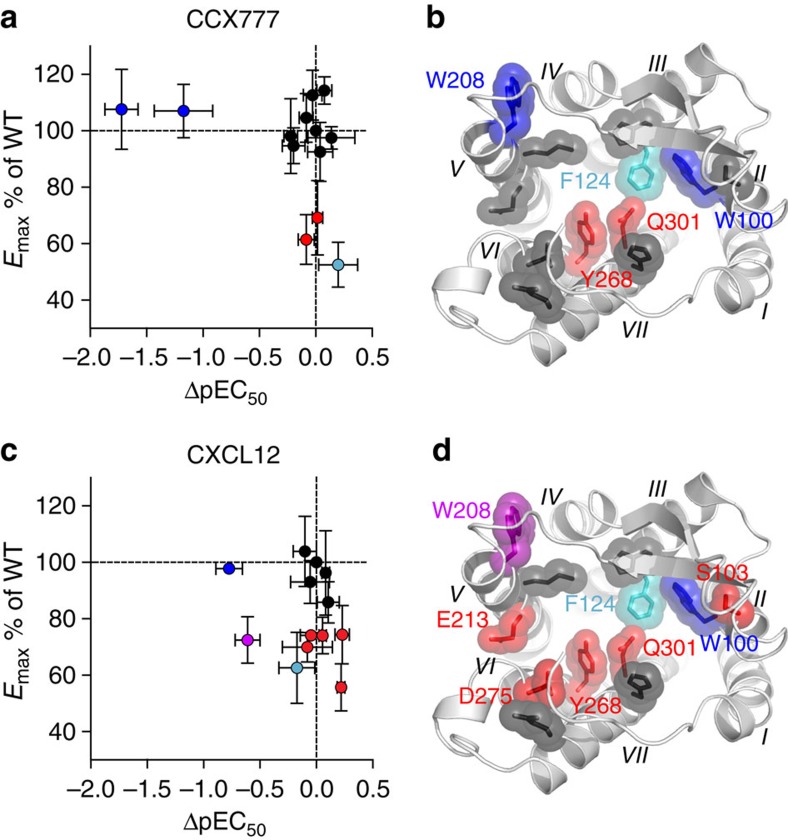
Functional characterization of orthosteric pocket mutants. (**a**,**c**) CCX777 and CXCL12 pEC_50_ and *E*_max_ values for ACKR3 mutants relative to WT-ACKR3. %*E*_max_ was determined as *E*_max,mutant_/*E*_max,WT_ × 100 and ΔpEC_50_ as pEC_50,mutant_−pEC_50,WT_. Each data point represents the average and standard error of the mean of three or more independent experiments. (**b**,**d**) Mutants mapped onto the orthosteric pocket of a CXCR4-based homology model of ACKR3. Residues are coloured according to the plots in (**a**) and (**c**), with blue and red corresponding to decreased pEC_50_ values or lowered *E*_max_, with respect to WT, respectively (*P*<0.05 from two-sided paired *t*-tests with *n*≥3). Residues that are unaffected by mutation are coloured in black, and W208, which has both decreased pEC_50_ and lowered *E*_max_ with CXCL12, shown in purple. F124A (cyan) is different from all other mutants in showing a reduced surface expression (60% of WT ACKR3) at virtually unchanged total expression (Supplementary Fig. 6). This may complicate the interpretation of the BRET data and contribute to the reduced *E*_max_ values. See Supplementary Table 5 for the identities, sample sizes and pEC_50_ and *E*_max_ values for all mutants.

To allow for the most robust comparison of mutant functional responses, all results are interpreted in the context of WT BRET measurements performed on the same day. Two mutations, W100^2.60^A and W208^5.34^A (Ballesteros-Weinstein numbering^35^ in superscript), reduce the potency of both CCX777 and CXCL12, consistent with the two ligands sharing overlapping binding interfaces (Fig. 6 and Supplementary Table 5). Interestingly, both W100^2.60^ and W208^5.34^ are protected by CCX777 as compared to CXCL12 in the radiolytic footprinting experiments (Fig. 4) and they are the only two residues that are protected among the 12 mutated sites. By contrast, mutation of M212^5.38^ on TM5 has no effect on β-arrestin-2 recruitment, which is also consistent with this residue being oxidized but not protected by compound or chemokine in the radiolytic footprinting experiments. In addition to the effect on potency, three mutants (F124^3.32^A, Y268^6.51^A and Q301^7.39^A) decrease the efficacy of CCX777-induced β-arrestin-2 recruitment as determined from the difference between BRET ratios at zero and saturating ligand concentrations (again with the caveat that F124A shows reduced surface expression). These positions are known to be part of the ligand-binding interface for several GPCRs including chemokine receptors. Moreover, a survey of all available GPCR structures revealed that residues at these positions make consensus contacts with ligands across all class A GPCRs^36^. The same residues along with four other sites (S103^2.63^A, W208^5.34^A, E213^5.39^A and D275^6.58^A) lower the efficacy of CXCL12-induced β-arrestin-2 recruitment, indicating that more ACKR3 residues are involved in activation by CXCL12 than the small-molecule CCX777.

### Experiment-guided 3D modelling of ACKR3 complexes

To interpret our results in a structural context, we utilized experiment-guided homology modelling and molecular docking to generate models of ACKR3 in complex with CCX777 and CXCL12. The overall flow of the ACKR3:CXCL12 modelling procedure involved positioning of the globular core of the chemokine with respect to the TM domain of the receptor, with temporary exclusion of the flexible N termini of both molecules, followed by flexible docking of the N-terminal peptides on their respective partners (described in refs 14, 33). To reconcile the radiolytic footprinting observations as well as the pairwise residue proximity restraints from the cysteine trapping experiments, the initial domain positioning required additional conformational sampling, with the result demonstrating significant differences when compared, for example, to the crystal structure of CXCR4:vMIP-II. Specifically, this sampling created an extensive binding interface between the receptor ECL2 and the region on the chemokine involving its proximal N terminus and the 30s loop, while at the same time maintaining interactions between the N terminus of ACKR3 and the N-loop of CXCL12 that are observed crystallographically in homologous receptor:chemokine pairs^14^,^16^.

**Figure 7:**
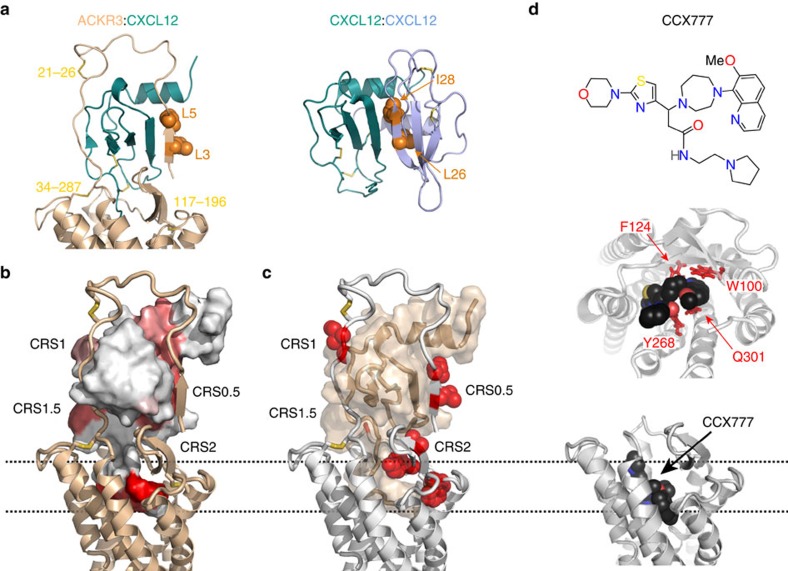
Ligand interactions in ACKR3 complexes. (**a**) The CRS0.5 interaction mimics the CXCL12 dimerization interface (PDB ID: 2k01)^30^. Side chains of homologous residues in ACKR3 (L3 and L5) and the second CXCL12 protomer (L26 and I28) are highlighted as orange spheres. (**b**) ACKR3:CXCL12 complex with the chemokine coloured shades of red according to protection in the ACKR3:CXCL12 complex as determined from radiolytic footprinting experiments. Darker red colour corresponds to stronger protection (see Fig. 3a). (**c**) Same complex as in (**b**). Side chains of receptor residues that are protected in the ACKR3:CXCL12 complex relative to the ACKR3:CCX777 complex (see Fig. 4a,b) are shown as red spheres. (**d**) Side and top views of the extracellular region of the ACKR3:CCX777 complex with CCX777 shown in black. Side chains of residues where mutation affects CCX777-induced β-arrestin-2 recruitment are shown as red sticks.

In line with the incomplete nature of the crystallographic templates^14^,^16^, both of which lack ∼20 distal N-terminal residues of the receptor, the initial ACKR3:CXCL12 models only contained receptor residues 21–318. To reconcile the radiolytic footprinting observations including protection of L3 and L5 in the distal N terminus of the receptor, as well as residues I28, L29, Y61 and L62 in the chemokine, we developed a structural hypothesis for the placement of the distal N terminus of the receptor on the chemokine in a region we call CRS0.5 by analogy with the existing nomenclature. The model predicts formation of an antiparallel β-sheet between the receptor CRS0.5 and the first β-strand of a CXCL12 monomer in a manner that mimics CXCL12 dimerization (Fig. 7a). This hypothesis is complementary to our discovery that CC chemokines also repurpose their dimer interface for receptor binding^14^. The predicted CRS0.5 interactions are fully consistent with the radiolytic footprinting experiments and are readily accommodated in the context of the proximal CRS1 interactions that were modelled by homology.

The resulting ACKR3:CXCL12 model (Fig. 7b,c) reveals an extensive binding interface that covers virtually the entire surface of the chemokine (consistent with radiolytic footprinting observations). The proximal CXCL12 N terminus interacts with ECL2 of ACKR3; the distal N terminus is completely buried in the orthosteric pocket of the receptor forming the CRS2 interaction site. The receptor N terminus runs antiparallel to the N-loop of CXCL12, making several interactions with the chemokine, including the side chains of I27 and V28 and residing in a hydrophobic patch involving V18 of the chemokine. The C21–C26 disulfide restricts a four-residue loop and is positioned at the junction of the CXCL12 N-loop and β_1_ strand, consistent with the CRS1 disulfide trapping results. Together, these contacts make up the CRS1 interactions. Residues 2–6 of ACKR3 form an antiparallel β-sheet with the β_1_ strand (residues 25–29) of CXCL12 (CRS0.5).

The small-molecule CCX777 was docked into the orthosteric pocket of ACKR3 in keeping with the results of our mutagenesis experiments and with SAR data of the compound series^37^. CCX777 resides in the orthosteric pocket of ACKR3 and makes several contacts with TM 3, 5, 6 and 7. Its core ring structures are anchored to the bottom of the pocket between residues W100^2.60^, F124^3.32^, Y268^6.51^ and Q301^7.39^ (Fig. 7d), which all affect CCX777 signalling when mutated to Ala. Mutation of W208^5.34^ affects both CXCL12- and CCX777-induced β-arrestin-2 recruitment, but this residue is not directly contacting the compound or the chemokine in the models. Moreover, this residue points away from the orthosteric pocket in all chemokine receptor and other GPCR structures^14^,^15^,^16^,^17^. Therefore, the effect of W208^5.34^A mutation on ligand-induced activation is likely allosteric. The CCX777 binding epitope is shared with small-molecule ligands from previously crystallized receptor:chemokine complexes (IT1t with CXCR4; ref. 14 and Maraviroc with CCR5; ref. 15). However, whereas IT1t primarily interacts in the ‘minor' side of the CXCR4 orthosteric pocket (TM 2, 3 and 7), CCX777 and Maraviroc both utilize the full binding pocket to interact with their respective receptors. As expected from site-directed mutagenesis, CCX777 overlaps with the N terminus of CXCL12 in the binding pocket, but lacks receptor:chemokine interactions at the top of the pocket and with ECL2.

### Detection of activation-dependent conformational changes

**Figure 8:**
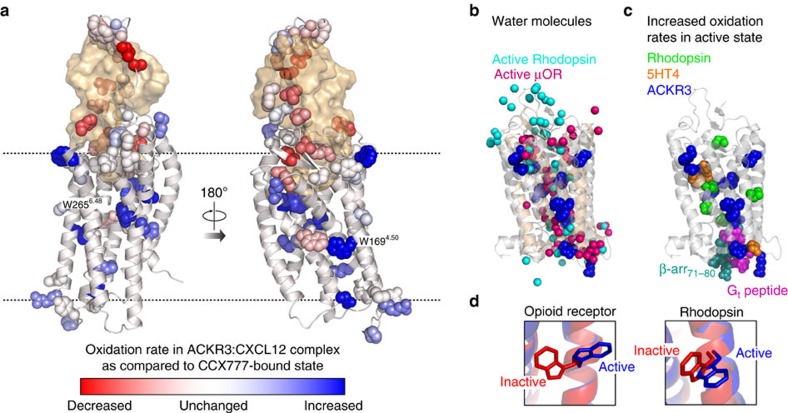
Activation of ACKR3 probed by radiolytic footprinting. (**a**) Mapping of radiolytic footprinting results on the ACKR3:CXCL12 complex. Side chains of all receptor residues with measured oxidation rates are shown as spheres and coloured based on the log of Rate_ACKR3:CCX777_/Rate_ACKR3:CXCL12_ using the same scheme as in Fig. 4. In the gradient colouring, blue corresponds to higher and red to lower oxidation rates in the ACKR3:CXCL12 (agonist) complex compared to the ACKR3:CCX777 (partial agonist) complex. (**b**) Overlay of waters from crystal structures of the μ-opioid receptor (μOR; backbone in wheat and waters in pink, PDB ID: 5c1m; ref. 43) and rhodopsin (backbone in gray, waters in cyan, PDB ID: 4x1h; ref. 41) in active conformations with residues that have increased oxidation rates in the ACKR3:CXCL12 complex. Side chains for residues with >40% higher oxidation rates in the ACKR3:CXCL12 complex are shown as blue spheres and mapped onto the rhodopsin structure. (**c**) Radiolytic footprinting studies of 7TM receptors mapped onto the crystal structure of active rhodopsin (PDB ID: 4x1h; ref. 41). Side chains of residues with increased oxidation rates in the active state of the receptor are shown as spheres for rhodopsin^23^ (Rho*-G_t_>Rho, cyan), 5HT4 (ref. 24) (apo>inverse agonist bound, orange), and ACKR3 (CXCL12-bound>CCX777-bound, blue). A G-protein-derived peptide is shown in magenta and residues 71–80 of β-arrestin from the rhodopsin:β-arrestin complex (PDB ID 4zwj; ref. 47) are shown as teal spheres. (**d**) Conformation of W^4.50^ side chain in active and inactive crystal structures of opioid receptors (PDB ID 5c1m (ref. 43) and 4n6h (ref. 45)) and rhodopsin (PDB ID 4zwj (ref. 47) and 1f88 (ref. 68)).

When compared to ACKR3:CCX777, radiolytic footprinting of ACKR3:CXCL12 reveals numerous residues in the extracellular domains and the orthosteric binding pocket of ACKR3 that are protected from oxidation, most likely due to direct shielding of the residues from solvent by the chemokine. However, we also observe oxidation rate differences for residues located in the transmembrane and intracellular region of ACKR3 that cannot be attributed to direct interaction with the ligands (Fig. 8a). These changes can only be explained by conformational rearrangements of the receptor in response to ligand binding, which in turn leads to changes in bulk solvent accessibility and/or coordination of structured water molecules within the TM domain^23^. The differences in the oxidation rates between the full (CXCL12) and partial (CCX777) agonist complexes are expected to report on fully activated and partially active receptor conformations, respectively.

When mapped onto the ACKR3:CXCL12 complex model, the residues whose oxidation rate increases in the full agonist complex form a defined path extending from the ligand-binding pocket through the conserved W265^6.48^ to the intracellular side of the receptor (Fig. 8a). Many of these residues are buried in the TM domain of the receptor and cannot possibly be exposed to bulk solvent; therefore, their changes in oxidation are due to rearrangement of structured water molecules within the TM domain. Such water molecules are consistently found in several 7TM receptors by X-ray crystallography or structure-based MS experiments^23^,^38^,^39^, and their positions appear to be affected by receptor activation, leading to restructuring of water-mediated polar residue networks^40^,^41^,^42^. The path of ACKR3 residues with agonist-induced increases in oxidation rates is in striking agreement with solvent networks identified in active states of both rhodopsin and opioid receptors (Fig. 8b)^41^,^43^. Previous radiolytic footprinting studies of GPCRs have shown that higher oxidation rates of residues lining this path correlate with an increased population of active receptor conformations both for rhodopsin (photoactivated and transducin-bound (Rho*−G_t_)>photoactivated (Rho*)>ground state (Rho)) and the serotonin type 4 (5-HT4) receptor (apo>inverse agonist bound)^23^,^24^ (Fig. 8c). Taken together, this suggests that as with other receptors, transition of ACKR3 toward more active conformations is accompanied by recruitment and ordering of water molecules in the transmembrane domain.

The network of residues with agonist-induced increase in oxidation includes W265^6.48^, which is known to be a crucial residue for GPCR activation^43^. In several GPCRs, a homologous residue has been shown to coordinate a sodium ion in the inactive state and to undergo a side-chain rearrangement on receptor activation^44^, consistent with our interpretation of its role in ACKR3. W169^4.50^ was identified as another residue with a higher oxidation rate in the ACKR3:CXCL12 complex (Fig. 4 and Supplementary Table 2). This residue is completely conserved among 7TM receptors and has been demonstrated to switch its rotameric state between inactive and active structures in some GPCRs including opioid receptors^43^,^45^ (Fig. 8d). On the intracellular side, the increased oxidation rates of residues D78^2.38^, P152^ICL2^ and R155^ICL2^ in the ACKR3:CXCL12 complex (Fig. 4 and Supplementary Table 2) suggest that in the active state, ACKR3 opens up to expose a binding site for intracellular signalling proteins. In line with this observation, GPCRs are known to undergo a conformational change on activation, involving an outward movement of TM6 to accommodate binding of G proteins and β-arrestins^40^,^46^,^47^.

Taken together, these observations support a similar structural mechanism for agonist-induced activation of ACKR3 and canonical GPCRs. However, the atypical nature of ACKR3 and its inability to couple to G proteins suggest that details of this mechanism must be different. Further studies are needed to provide sufficient resolution for identification of these details, and their elucidation remains the subject of future structure determination efforts.

## Discussion

In this study, we investigate the structural basis of the interaction between ACKR3 and its endogenous chemokine agonist, CXCL12, as well as with the small-molecule partial agonist, CCX777, using a synergistic combination of radiolytic footprinting, disulfide trapping, mutagenesis, functional experiments and molecular modelling. The models and experimental data reveal both similarities and unique features of ACKR3 compared to G-protein-coupled chemokine receptors^14^,^15^,^16^.

The proposed ACKR3:CXCL12 model is consistent with common interaction architecture proposed for all/most receptor:chemokine complexes and involves two main interaction epitopes: (i) CRS1 where the N terminus of the receptor binds to the N-loop and 40s-loop of the chemokine and (ii) CRS2 where the N terminus of the chemokine interacts with ECL2 and the TM domain pocket of receptor. In addition to confirming this architecture, the recent crystal structures of CXCR4:vMIP-II and US28:CX3CL1 highlighted an unexpected intermediate region that we termed CRS1.5^14^; it involves the formation of an antiparallel β-sheet between the proximal N termini and the conserved cysteines of the receptor and the chemokine. Although chemokines bind their receptors as monomers, they oligomerize^48^, and interestingly, the CRS1.5 interface mimics the oligomerization interfaces of CC and CX3C chemokines^49^,^50^. CXC chemokines, on the other hand, dimerize through interactions involving the β_1_ strand, forming a six-stranded antiparallel β-sheet (Fig. 7a). In this study, radiolytic footprinting confirms that residues L3 and L5 of the ACKR3 N terminus and residues I28 and L29 in the β_1_ strand of CXCL12 are protected, and modelling suggests that they form an antiparallel β-sheet interaction that mimics the CXCL12 dimer interface (Fig. 7a). These interactions, referred herein as CRS0.5, are likely present in other CXC receptor:chemokine complexes but the dynamic nature of the receptor N terminus may make their detection in crystal structures rather challenging^14^,^16^. For different CXC receptor:chemokine pairs, interactions in this region can explain a number of experimental observations that could not be explained by previous structures and models: (i) cross-saturation NMR experiments suggested that the β_1_ strand of CXCL12 is in close proximity to the CXCR4 N terminus^51^, (ii) truncation of residues 2–9 of CXCR4 affects both CXCL12 binding and activation of CXCR4 (ref. 52), (iii) D10 is critical for CXCL12-induced activation of CXCR4 (ref. 53), (iv) sulfation of residues Y7 and Y12 of CXCR4 affects CXCL12-induced signalling^54^, (v) nuclear Overhauser effects have been measured in this region between CXCL12 and a peptide corresponding to residues 1–38 of the CXCR4 N terminus^55^, (vi) mutation of CXCR2 residues 7 and/or 9 affects chemokine binding and signalling^56^ and (vii) mutations in the β1 strand of CXCL8 reduce its affinity for CXCR1 (ref. 57). Our detection of the CRS0.5 interactions emphasizes the need to combine diverse approaches to understand the structure and dynamics of 7TM receptors. Radiolytic footprinting and other solution techniques such as NMR and H/D exchange can capture dynamic processes related to ligand-binding and receptor activation, making them highly complementary to the more static pictures obtained from receptor:chemokine crystal structures.

While possessing the conserved fold and chemokine complex architecture, ACKR3 has a number of unique features. One of them is an intramolecular disulfide bridge connecting C21 and C26 in the ACKR3 N terminus. Further experiments are needed to fully understand the functional importance of this disulfide but it appears highly conserved across species. Two other human chemokine receptors, CXCR3 and CCR7, have pairs of Cys residues in their extracellular domains. To the best of our knowledge, disulfide formation has not yet been proposed or confirmed for either of these receptors. However, in analogy with ACKR3, the Cys residues likely form a disulfide in the oxidizing extracellular environment. Interestingly, ACKR3 and CXCR3 both interact with the chemokine CXCL11, suggesting that the disulfide motif could be a recognition determinant for this chemokine.

As an atypical receptor, ACKR3 exclusively couples to β-arrestin and does not signal through G proteins. This is despite the fact that it possesses the hallmark G-protein signalling motifs such as the DRY sequence at the end of TM3 and the NPxxY motif in TM7. As such, ACKR3 represents a unique system for studying the structural basis of 7TM receptor bias.

This study identified numerous similarities between ACKR3 and canonical GPCRs; for example, the residues in the orthosteric pocket that affect ACKR3 activation (β-arrestin recruitment) are homologous to those that affect G-protein signalling in other receptors. Additionally, we demonstrate that the conformational changes that ACKR3 undergoes on agonist binding are strikingly similar to those on activation of canonical GPCRs such as rhodopsin and μ-opioid receptor, which suggests that the conformational control of bias is subtle. For example, ^19^F-nuclear magnetic resonance^58^ studies of β_2_ adrenergic receptor located unique conformational changes associated with β-arrestin biased signalling to the C-terminal end of TM7; this could be correlated to the increased radiolytic oxidation rates of ACKR3 TM7 residues C308^7.46^ and C309^7.47^ in the presence of CXCL12. Furthermore, the bias towards β-arrestin signalling could be governed by other (non-conformational) mechanisms such as ligand kinetics, receptor distribution in the membrane, or post-translational modifications.

In recent years, biased 7TM receptor ligands that preferentially activate either G proteins or β-arrestin pathways gained interest as novel therapeutics^59^. However, the structural basis of biased agonism remains poorly understood, partly because the available crystal structures of G-protein- and β-arrestin complexed GPCRs^41^,^46^,^47^ do not show any pronounced conformational differences in the receptors when compared with each other. The present study adds to the growing body of evidence supporting the similarity of receptor conformational changes that lead to activation of G-protein- versus β-arrestin-mediated pathways. Additionally, as the first structural mapping of an atypical chemokine receptor and the most thorough radiolytic footprinting mapping of a 7TM receptor to date, this study dramatically expands current knowledge of the structural determinants of ligand-binding and ligand-induced signalling in chemokine and other 7TM receptors.

## Methods

### Production of recombinant proteins in *E. coli*

Recombinant CXCL12 was expressed and purified from *E. coli.* A pET21-based vector containing residues 22–89 of human CXCL12α (GenBank accession code NM_199168.3) preceded by an enterokinase recognition site and a His_8_ tag was transformed in BL21(DE3)pLys cells (Promega). Cells were grown to an optical density of 0.7 in Luria-Bertani (LB) medium, protein expression was induced by the addition of 0.5 mM isopropyl β-D-1-thiogalactopyranoside (IPTG) and 6 h after induction cells were harvested by centrifugation. Cell pellets were suspended in lysis buffer (50 mM Tris, pH 7.5, 150 mM NaCl) and lysed by sonication. The lysate was centrifuged (6,000 *g* for 15 min) and the pellet containing CXCL12 inclusion bodies was dissolved in 50 mM Tris, pH 8, 6 M guanidine-HCl, 4 mM DTT (dithiothreitol). The resulting suspension was sonicated and centrifuged (6,000 *g* for 15 min) and the supernatant was passed over a Ni-NTA agarose resin (Qiagen) using gravity flow. The resin was washed with 50 mM MES, pH 6, 6 M guanidine-HCl, 4 mM DTT and protein was eluted with 50 mM acetate, pH 4, 6 M guanidine-HCl and 4 mM DTT. The eluate was diluted 10-fold with refolding buffer (50 mM Tris, pH 7.5, 500 mM arginine-HCl, 1 mM EDTA, 1 mM glutathione disulfide) and incubated for 60 min with stirring. The refolding mixture was dialyzed in 20 mM Tris, pH 8.0 and 50 mM NaCl. After dialysis, 2 mM CaCl_2_ was added and the His_8_ tag was cleaved through the addition of enterokinase (New England Biolabs). After cleavage the mixture was added to a Ni-NTA resin and cleaved CXCL12 was eluted with 6 M guanidine and 50 mM MES, pH 6.0. The eluate was bound to a reversed-phase C18 HPLC column (Vydac) (buffer A: 0.1% trifluoracetic acid; buffer B: 0.1% trifluoroacetic acid; 90% acetonitrile) and eluted by linearly increasing the buffer B concentration from 33 to 45%. The resulting pure CXCL12 protein was lyophilized and stored at −80 °C until use.

HRV3C protease was produced in *E. coli*. A plasmid containing the HRV3C sequence and an N-terminal His tag was transformed into BL21(DE3)pLys cells and plated onto kanamycin-resistant LB agar plates. A single colony was picked and grown overnight in 10 ml LB media supplemented with 30 μg ml^−1^ kanamycin at 37 °C. The overnight culture was diluted 1:100 in LB/kanamycin and grown to an OD_600_ of 0.6–0.8, at which point protein expression was induced through the addition of 0.5 mM IPTG. At 3 h after inducation, the cells were harvested by centrifugation. Cell pellets were resuspended in 50 mM Tris, pH 8.0, and sonicated for 10 min on ice. The suspension was then centrifuged (50,000 *g* for 20 min) and pellets were homogenized in lysis buffer (100 mM Na_2_H_2_PO_4_, 10 mM Tris, 6 M urea, pH 8.0). The resulting suspension was centrifuged (50,000 *g* for 25 min) and the supernatant was incubated for 1 h with Ni-NTA resin. The resin was washed with lysis buffer and protein was eluted with elution buffer (50 mM sodium acetate, 6 M urea, pH 5.0). The eluted sample was then dialyzed twice against dialysis buffer (20 mM MES, 1 mM EDTA, 1 mM DTT, 10% (v v^−1^) glycerol, pH 6.5), flash frozen and stored at −80 °C until further use.

### Expression of receptors and chemokines in Sf9 cells

For expression in Sf9 cells (ATCC), residues 22–89 of human CXCL12α (GenBank accession code NM_199168.3) were cloned into a pFastBac1 vector containing a polH promoter to drive protein expression (see Supplementary Table 6 for a list of all primers used in this study). Residues 2–362 of human ACKR3 (GenBank accession code NM_020311.2) were cloned into a pFastBac1 vector containing a GP64 promoter, HA signal sequence and C-terminal FLAG and His tags using *Asc*I and *Fse*I cloning sites. Cys mutants of ACKR3 and CXCL12 were produced using standard site-directed mutagenesis methods.

Baculovirus stocks containing the different ACKR3 and CXCL12 constructs were produced using the Bac-to-Bac Baculovirus Expression System (Invitrogen) as described previously^20^. Briefly, ACKR3 and CXCL12 pFastBac1 vectors were transformed into DH10Bac cells (Thermo Fisher) and spread onto LB agar plates with 50 μg ml^−1^ kanamycin, 7 μg ml^−1^ gentamicin, 10 μg ml^−1^ tetracycline, 100 μg ml^−1^ Bluogal and 40 μg ml^−1^ IPTG (Teknova). Single colonies were inoculated overnight in 5 ml LB media containing 50 μg ml^−1^ kanamycin, 7 μg ml^−1^ gentamicin and 10 μg ml^−1^ tetracycline; cells were pelleted by centrifugation. The pellets were resuspended in cold buffer P1 (Qiagen), lysed with buffer P2 (Qiagen) and neutralized by buffer P3 (Qiagen). Samples were centrifuged (10 min 15,000 *g*), the supernatant was transferred to a new tube, 700 μl isopropanol was added and samples were incubated for 15 min on ice. Bacmids were pelleted by centrifugation (10 min 15,000 *g*), washed with 70% ethanol and solubilized in 40 μl of 10 mM Tris, pH 8 and 1 mM EDTA. Five microlitres of purified bacmid was combined with 3 μl of X-tremeGENE HP DNA and 100 μl of transfection medium (Expression Systems) and incubated for 30 min. The mixture was added to 2.5 ml of Sf9 cells at 1.2–1.4 × 10^6^ cells ml^−1^ and cells were incubated for 96 h at 27 °C and 300 r.p.m. shaking. After 96 h, the cells were centrifuged (10 min 2,000 *g*) and 400 μl of the supernatant (P0 virus) was added to 40 ml of Sf9 cells at a density of 2.2–2.6 × 10^6^ cells ml^−1^. Cells were incubated at 27 °C and 130 r.p.m. shaking for 48 h, centrifuged (10 min 2,000 *g*) and the supernatant (P1 virus) was stored at 4 °C until use. Protein expression was achieved by adding ACKR3 and CXCL12 P1 virus (for ACKR3:CXCL12 samples) or ACKR3 P1 virus (for ACKR3:CCX777 samples) to Sf9 cells at a density of 2.5 × 10^6^ cells ml^−1^ and a multiplicity of infection of 5. After 48 h, cells were harvested by centrifugation and cell pellets were stored at −80 °C.

### Preparation of receptor complexes

Cell pellets were thawed and homogenized in hypotonic buffer (10 mM HEPES, pH 7.5, 20 mM KCl, 10 mM MgCl_2_, EDTA-free protease inhibitor mixture (Roche)) using 30 strokes in a Dounce homogenizer. After centrifugation at 50,000 *g*, the process was repeated once in hypotonic buffer and three times in high salt buffer (hypotonic buffer+1 M NaCl). The final membrane pellet was homogenized in hypotonic buffer containing 30% (v v^−1^) glycerol (25 ml l^−1^ culture), frozen and kept at −80 °C until further use. The membrane suspension was thawed on ice, diluted with equal volume of hypotonic buffer and incubated with 2 mg ml^−1^ of IA. For ACKR3:CCX777 samples, 100 μM CCX777 (Chemocentryx Inc.)^37^ was added to the buffer. After 30 min of incubation at 4 °C, the sample volume was doubled by the addition of 2 × solubilization buffer (100 mM HEPES, pH 7.5, 800 mM NaCl, 1.5/0.3% (w v^−1^) DDM/CHS) and incubated for 3h. The sample was centrifuged to remove insoluble material and the supernatant was incubated with TALON IMAC resin (Clontech) (2 ml of 50% slurry per litre culture) and 10 mM of imidazole overnight. Samples were then centrifuged for 5 min at 350 *g*, supernatants were discarded and the resin was transferred to gravity flow columns. After washing with 20 column volumes of wash buffer 1 (25 mM HEPES, pH 7.5, 400 mM NaCl, 10% glycerol, 0.1/0.02% DDM/CHS, 10 mM imidazole) and 10 column volumes of wash buffer 2 (25 mM HEPES, pH 7.5, 400 mM NaCl, 10% glycerol, 0.025/0.005% DDM/CHS, 10 mM imidazole), protein was eluted with three column volumes of elution buffer (wash buffer 2 with 250 mM imidazole).

For disulfide crosslinking experiments, the eluted protein solution was exchanged into buffer exchange buffer (wash buffer 2 without imidazole) using 0.5 ml 100 kDa molecular weight cutoff spin concentrators. Samples were loaded onto non-reducing 10% SDS–PAGE gels and crosslinked complexes were detected based on their increased molecular weight and comigration with chemokine using Coomassie staining and western blotting. Mouse anti-Flag M2 primary antibody (1:5,000 dilution, F3165; Sigma Aldrich) and IRDye 680-conjugated donkey anti-mouse IgG (1:20,000 dilution; LI-COR Biosciences) secondary antibody were used to detect the FLAG-tagged receptor. HA-CXCL12 was detected using a rat anti-HA 3F10 primary antibody (1:5,000 dilution, 11867423001; Roche) and IRDye 800-conjugated goat anti-rat IgG (1:20,000 dilution; LI-COR Biosciences) secondary antibody.

For radiolytic footprinting, SDS–PAGE, SEC and thermal unfolding experiments, the eluted receptor was concentrated to 500 μl using 15 ml 100 kDa molecular weight cutoff spin concentrators. The concentrated samples were then applied to a PD-10 desalting column (GE Healthcare) to remove imidazole. The column was equilibrated with 6 ml of radiolytic footprinting buffer (25 mM cacodylate, pH 7, 150 mM NaCl, 0.025/0.005% DDM/CHS) and the flow-through was discarded. One microlitre of radiolytic footprinting buffer was added to the column and the protein-containing flow-through was collected. HRV3C protease (produced as described above) and PNGaseF (New England Biolabs) were added and the sample was incubated for 16 h at 4 °C. 500 μl of 50% TALON slurry and 3 mM imidazole was added; the sample was incubated for 90 min and then transferred to a gravity flow column. The column flow-through was collected and concentrated to 1 mg ml^−1^ to use for characterization (SDS–PAGE, SEC and thermal unfolding) and radiolytic footprinting experiments.

For thermal unfolding experiments, 0.3 μM of protein (ACKR3:CCX777 or ACKR3:CXCL12) was incubated with 2.5 μM of CPM (7-diethylamino-3-(4′-maleimidylphenyl)-4-methylcoumarin) dye in radiolytic footprinting buffer. The temperature was ramped from 25 to 95 °C and CPM fluorescence (excitation 365 nm, emission 460 nm) was measured using a RotorGene Q 6-plex RT-PCR machine (Qiagen). For SEC experiments, a Sepax SRT-C 300 column was equilibrated with radiolytic footprinting buffer, 10 μg of ACKR3:CCX777 or ACKR3:CXCL12 was loaded onto the column at a flow rate of 0.5 ml min^−1^ and elution of the protein was detected using absorbance at 280 nm.

### Synchrotron X-ray radiolysis

Synchrotron X-ray radiolysis was performed at beamline X28C of the National Synchrotron Light Source. ACKR3:CXCL12, ACKR3:CCX777 and CXCL12 samples in 25 mM cacodylate, pH 7.2, 150 mM NaCl and 0.025/0.005% DDM/CHS were exposed to X-ray using conditions that were optimized based on a standard fluorophore assay, which measures the decay of Alexa 488 intensity^60^. Alexa dose–response curves were generated for all three samples to measure accurately the level of dose and/or scavenging in the samples. In these experiments, Alexa 488 is spiked into the protein samples and loss of fluorescence as a function of X-ray dose is determined. Similar rate constants for the radiolytic degradation of Alexa was observed in all three samples (53, 50 and 49 s^−1^ for ACKR3:CXCL12, ACKR3:CCX777 and CXCL12, respectively), demonstrating that they all experienced similar synchrotron radiolysis conditions and are thus directly comparable without corrections. A two-step modified KinTek (KinTek Corp.) apparatus was applied for delivering protein samples during exposure. First, 200 μl of the sample was allowed to flow through a 3.5 μl cell for irradiation at rates ranging from 0 to 15 ms. To quench secondary oxidation by excessive free radicals, methionine/amide solution was added in the flow within 40 ms so that samples were collected in 10 mM methionine/amide solution in the second step. The procedures were performed at 10 °C. Exposed samples were frozen in dry ice and stored at −80 °C.

### Proteolysis

Duplicate X-ray-exposed samples were digested by pepsin and trypsin (Promega)/AspN (Roche) to obtain high-sensitivity detection of both membrane domain and soluble regions (N terminus, C terminus and loops). Before proteolysis, samples (∼200 μl) were loaded into a 3K centrifugal filter (Amicon Ultra). Four hundred microlitres of 8 M urea containing 80 mM DTT was added to reduce disulfide bonds and the samples were incubated at 37 °C for an hour. Samples were then centrifuged at 1,400 *g* for 15 min, 200 μl of 8 M urea was added to the filter to wash away detergent and small molecules and the centrifugation was repeated. Two hundred microlitres of 8 M urea containing 25 mM IA was added and the samples were incubated at 37 °C for an hour to block free cysteines. Reduced protein samples were washed once with 400 μl of 1% formic acid and once with 400 μl of 0.1% formic acid to remove small molecules. Samples were concentrated to ∼25 μl by spinning at 1,400 *g* for 15 min.

When pepsin was used for protein digestion, 10 μl of 20 ng μl^−1^ pepsin was added to samples in 0.1% formic acid (pH ∼2.5), and incubated overnight. When trypsin/AspN was used for protein digestion, samples were vacuum-dried and reconstituted with 10 μl of 20 ng μl^−1^ trypsin in 100 mM Tris buffer (pH ∼8). After incubating overnight, samples were heated to 70 °C for 5 min and then cooled down to room temperature. Five microlitres of 40 ng μl^−1^ AspN solution was then added to the samples and incubated overnight, followed by 1 μl of 50% formic acid to quench the digestion.

### Disulfide bond identification

For disulfide bond detection, protein was digested by pepsin or a combination of LysC (Wako Chemicals USA), trypsin (Promega) and AspN (Roche). Briefly, 5 μl of 1 μg μl^−1^ ACKR3 sample was denatured and free cysteines blocked by the addition of 400 μl of 8 M urea containing 100 mM IA for 1 h. Samples were washed with 400 μl of 8 M urea with no IA and 400 μl of 1 M urea two times on a 3K centrifugal filter (Amicon Ultra). Samples were collected by flipping tubes and centrifuged at 1,000*g* for 1 min. The enzyme to protein ratio for digestion was 1:20. Pepsin digestions were performed in 0.1% formic acid overnight. For LysC/trypsin/AsnN protease digestion, LysC was used to digest protein for 4 h at 37 °C, followed by trypsin digestion at 37 °C overnight. AspN was then added for an additional incubation overnight at 37 °C.

### Nano-electrospray ionization-MS/MS

MS experiments were carried out with an Orbitrap Elite hybrid mass spectrometer (Thermo Finnigan, San Jose, CA, USA). Nano-reversed-phase liquid chromatography separations were performed on a UPLC (Waters, Milford, MA, USA) with a 5 cm × 75 μm Pico Frit C18 column (New Objective, Woburn, MA, USA) directly connected to a nanospray emitter (10 μm; New Objective).

For tryptic digests, chromatography was performed by using mobile phases A (0.1% formic acid in water) and B (100% acetonitrile, 0.1% formic acid in water) with a 90min nonlinear gradient at a flow rate of 0.3 μl min^−1^. The gradient started with 1% mobile phase B and was gradually increased to 15% for 21 min, and then to 25% for 56 min (22–78 min). After 79 min, phase B was changed to 90% and kept constant for 10 min. For peptic digests, chromatography was performed with a 2 h linear gradient starting with 1% and increasing to 40% phase B for 2 h. All data were acquired in positive ion mode. For these experiments, full MS scans (*m/z* 300–2,000) were followed by MS2 scans of the 10 most abundant peptide ions at a normalized collision energy of 35%. For detection of disulfide bonds in ACKR3, HCD cleavage mode was used to increase identification accuracy, as it provides relatively high-resolution MS2 (15,000) data compared to much lower resolution data for CID. High mass accuracy FT/MS was performed to detect precursor ions (resolution, 60,000; mass accuracy, 5 parts per million (p.p.m.)); product ions were detected in an ion trap with relatively low mass accuracy (1 Da).

### Data analysis

Tandem MS data were searched with the bioinformatics software MassMatrix^61^. The ACKR3 sequence and its reversed sequence were used as a database for identifying peptides from the Tandem MS data. In addition to built-in modifications such as deamidation of Asn and carbamidomethylation of Cys residues, all possible footprinting oxidations were enabled as variable modifications in the data analysis. *In silico* digestion was performed by either nonspecific cleavage when pepsin was used as the enzyme or cleavage after Arg, Lys and before Asp when trypsin and AspN were used as enzymes. A mass accuracy of 10 p.p.m. was set for searching precursor ions and 1 Da for product ions. For disulfide bond identification, crosslinks of disulfide bonds between cysteine residues was selected. Oxidation of methionine, labeling of cysteine (IA modifications) and deamidation of asparagine and glutamine were selected as variable modifications. All of the identified target proteins had scores higher than 1,000. The threshold limit of PP scores of identified peptides was set to be >5.0 (ref. 62). All of the detected modified peptide mass spectra were confirmed manually.

### Calculation of oxidation rates

The unmodified fraction of each peptide was calculated as the chromatographic peak area of the unoxidized species divided by the total peak areas of oxidized and unoxidized species. Unmodified fractions of the peptide were plotted against synchrotron exposure times to produce dose–response curves. Oxidation rates were then determined by the pseudo-first-order reaction equation: *Y*=*Y*_0_·e^−*kt*^ (where *Y* and *Y*_0_ are the fractions of unmodified peptide at time *t* and 0 ms, and *k* is the first-order rate constant). Data from two replicates of the sample were analysed statistically using Origin 9.0. Oxidation rates and standard deviations were calculated by fitting data from two replicates to the first-order equation.

### PFs and SASA calculations

PFs for single residues (or multiple residues within a peptide) were calculated by the dividing the intrinsic reactivity of the residue (or the sum of the intrinsic reactivities for all of the residues) by the measured oxidation rate constants as shown in equation (1),^27^ where the intrinsic reactivity data are from the website http://www.theyanglab.org/protection.html. The value of the intrinsic reactivity of each amino acid is derived from radiolysis data^63^ as


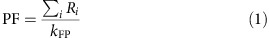


Here *R*_*i*_ is the intrinsic reactivity for each amino-acid residue and *k*_FP_ is the oxidation rate of the residue/peptide measured in the radiolytic footprinting experiment. The SASA of each residue was calculated based on the crystal structure of free chemokine CXCL12 (PDB ID: 1a15) using ICM^64^ by setting a probe of 1.4 for all atoms of each residue. A weighted SASA of a peptide was used to calculate the contribution of multiple residues in the peptide as given by the equation:


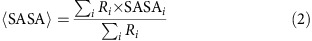


where SASA_*i*_ is the SASA of residue *i*. fSASA was calculated as the ratio between the observed weighted SASA and its residue-specific standard accessible area (SASA_REF_)^29^ using equations (3) and (4):


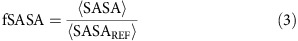



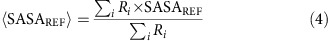


A Pearson's correlation coefficient (*R*) between the natural log of PF and fractional SASA for free CXCL12 was calculated by linear regression.

### BRET assay

The full human ACKR3 sequence, with an N-terminal HA tag, was cloned into a pcDNA vector containing the *Rluc3* gene (a kind gift from Nikolaus Heveker, Université de Montréal, Montreal, QC, Canada) such that the Rluc3 sequence was fused, after a short intervening linker, to the C terminus of the receptor. Single residue Ala mutations of ACKR3 were introduced using QuikChange mutagenesis (Agilent). Cells were obtained authenticated and guaranteed mycoplasma free from ATCC. HEK293S cells (ATCC) stably expressing GFP10-β-arrestin-2 (the pcDNA vector encoding GFP10-β-arrestin-2 was also a kind gift from Nikolaus Heveker, Université de Montréal) were maintained in Dulbecco's modified Eagle's medium supplemented with 10% fetal bovine serum and 700 μg ml^−1^ of G418. Cells cultured in 6-well plates were transiently transfected with 0.15–0.3 μg of ACKR3-Rluc3 DNA per well using TransIT-LT1 (Mirus). At 24 h after transfection, the cells were washed with PBS buffer and resuspended in BRET buffer (PBS buffer with 0.1% glucose). A total of 1 × 10^5^ cells were aliquoted into each well of a 96-well white clear-bottom tissue-culture assay plate (BD Falcon) and incubated for 45 min at 37 °C. Serial dilutions of CXCL12 and CCX777 were prepared in BRET buffer and added to the cells in triplicate. The plate was then incubated for an additional 15 min at 37 °C. GFP10-β-arrestin-2 expression was quantified by measuring GFP10 fluorescence with a SpectraMax M5 fluorescent plate reader (Molecular Devices), using excitation and emission wavelengths of 400 and 510 nm, respectively. The luciferase substrate Deep Blue C was added to a final concentration of 5 μM and BRET was measured immediately on a VictorX Light multilabel plate reader (Perkin-Elmer Life Sciences) as the ratio of GFP10 emission to Rluc3 emission, measured at 515 and 410 nm, respectively. *E*_max_ and pEC_50_ values were determined from nonlinear fitting of dose–response curves in GraphPad Prism. For each mutant, %*E*_max_ (*E*_max,mutant_/*E*_max,WT_ × 100) and ΔpEC_50_(pEC_50,mutant_−pEC_50,WT_) were determined utilizing data for WT-ACKR3 acquired on the same day as a reference. Averaged ΔpEC_50_ and %*E*_max_ and standard errors were calculated from three or more independent experiments performed on different days.

### Surface expression assay

Surface expression of HA-ACKR3-Rluc3 mutants was quantified using flow cytometry measurements. HEK293S cells stably expressing GFP10-β-arrestin-2 were transfected with 0.2 μg receptor DNA per well of a 6-well plate as described above for BRET assays. After 24 h, the cells were washed with PBS buffer and resuspended in PBS buffer with 0.5% bovine serum albumin (BSA). A total of 5 × 10^4^ cells per well were aliquoted into 96-well plates and staining was carried out in a 11 × dilution of anti-HA-APC (Miltenyi Biotec) (Clone GG8-1F3.3.1) for 20 min on ice. After staining, the cells were washed three times in PBS buffer with 0.5% BSA and fixed with 0.67 % paraformaldehyde. After fixing, the cells were washed once and resuspended in PBS buffer with 0.5% BSA. Data were acquired using a Guava bench top miniflow cytometer (Millipore) and analysed in FlowJo.

### Molecular modelling

Models of the ACKR3:CXCL12 complex were built in the ICM software package^64^. A hybrid modelling template was first built from the CXCR4 molecule in the CXCR4:vMIP-II structure (PDB 4rws; ref. 14) and a CXCL12 structure (PDB ID 3gv3; ref. 65) after rigidly superimposing the backbone atoms of CXCL12 residues C11, R12, V49 and C50 onto the corresponding atoms in vMIP-II (the core of the CRS1 interface), and after deleting the chemokine N terminus (residues 1–6). An initial model was built by threading the ACKR3 sequence into the CXCR4 template coordinates according to the sequence alignment. For stretches of <12 residues that aligned against gaps in the template, the algorithm first searched a library of PDB fragments for candidate conformations and then optimized them by extensive conformational sampling. The N termini of the receptor (residues 1–27) and the chemokine (residues 1–6) were omitted from this procedure. Residue side chains in the complex models were refined with 5 × 10^5^ steps of Monte Carlo optimization in internal coordinates. To reconcile the radiolytic footprinting observations as well as the pairwise residue proximity restraints from the cysteine-trapping experiments, the relative position of the receptor domain involving helices 2–5 (and ECLs 1 and 2) was additionally sampled, as a rigid body with flexible side chains, with respect to the receptor helices 1, 6 and 7, and the chemokine.

For chemokine N terminus refinement, the receptor was converted into a set of interaction potentials precalculated on a three-dimensional (3D) grid, including the potentials for van der Waals, electrostatic, hydrogen bonding and apolar surface interactions^66^. The chemokine N terminus (up to the second N-terminal cysteine residue) was built *ab initio*, its CXC motif tethered to the positions of the corresponding atoms in the template, and the N terminus thoroughly sampled in the receptor potential grids. The obtained stack of N terminus conformations was merged with the full-atom model of the receptor, and another 10–20 × 10^6^ steps of Monte Carlo optimization were performed, this time using full-atom receptor representation with flexible binding pocket side chains.

For receptor N-terminus refinement, the chemokine was represented as a set of 3D grid interaction potentials. The receptor N terminus (residues 1–34) was built *ab initio*, an intramolecular disulfide bond imposed (C21–C26), the conserved cysteine (C34) tethered to the positions of the corresponding residue in the template and the distal N terminus (residues 2–6) tethered to the β_1_ strand of the chemokine dimer partner from the PDB 3gv3 (residues H25:L29). The N terminus was then thoroughly sampled in the chemokine potential grids. The obtained stack of conformations was merged with the full-atom model of the chemokine, and another 10^8^ steps of Monte Carlo optimization were performed, this time using full-atom chemokine representation with flexible interface side chains.

For final model assembly, top-scoring conformations of receptor N terminus and the chemokine N terminus were merged with the remaining parts of both molecules into an intact complex, after which another short round of side-chain refinement was performed to remove residue clashes resulting from the merge.

Compound docking was performed using full-atom-biased Monte Carlo sampling of the ligand and the receptor binding pocket side chains in internal coordinates, as implemented in ICM^64^. Guided by the extensive SAR of CCX777 compound series^37^, the acceptor centres on the quinoline and the thiazole moieties and the donor on the azepane ring were tethered to the side chains of Q301 and Y268 in two alternative orientations. The rest of the compound and the surrounding binding pocket side chains were allowed to freely change their positions/conformations throughout the sampling procedure. Four candidate stereoisomers of the compound were generated before docking. The conformers of the saturated rings were sampled explicitly during docking. Sampling was performed to convergence. Following the sampling phase, top-ranking predicted poses of the ligands were re-evaluated using full-atom representation of the receptor pocket and the ICM ligand-binding score^67^ and the top-scoring pose was chosen.

### Data availability

The sequences of ACKR3 and CXCL12α proteins used in this study can be accessed via GenBank accession codes NM_020311.2 and NM_199168.3, respectively. The following PDB codes were used in this work: 1a15 (for CXCL12 SASA calculation), 3gv3 and 4rws (for ACKR3:CXCL12 complex homology modelling) and 2k01, 3sn6, 4x1h, 4zwj, 5c1m, 4n6h, 1f88, 4rws, 4xt1 (for comparative analysis). Coordinates of the ACKR3:ligand complex models are available on request from the authors. All other relevant data supporting the findings of this study are either provided in the Article and Supplementary files or available from the authors on request.

## Additional information

**How to cite this article:** Gustavsson, M. *et al*. Structural basis of ligand interaction with atypical chemokine receptor 3. *Nat. Commun.* **8,** 14135 doi: 10.1038/ncomms14135 (2017).

**Publisher's note:** Springer Nature remains neutral with regard to jurisdictional claims in published maps and institutional affiliations.

## Supplementary Material

Supplementary Information Supplementary figures and supplementary tables.
