# Supplementary material for: Structural basis of ligand interaction with atypical chemokine receptor 3
Source: Nat Commun. 2017 Jan 18;8:14135. doi: 10.1038/ncomms14135 (PMC5253664; doi:10.1038/ncomms14135)
Supplement: Supplementary Information — Supplementary figures and supplementary tables. [file ncomms14135-s1.pdf]

Supplementary information

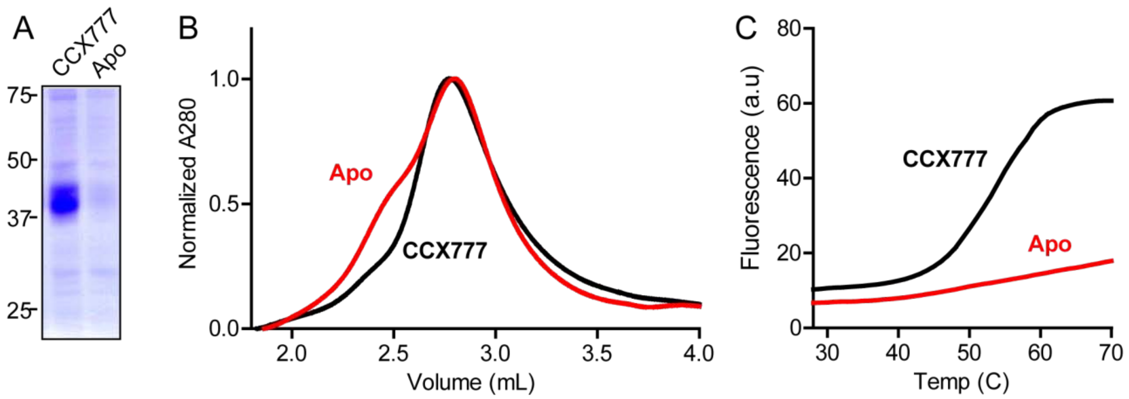

Supplementary Figure 1. Purification and characterization of *apo* vs. ligand-bound ACKR3. (**a**) Representative SDS-PAGE showing the yield of ACKR3 extracted with CCX777 or without ligand. (**b**) SEC traces of the samples from (a), normalized to 100% of maximum absorbance. The shoulder of the apo peak (eluting around 2.5mL) indicates that the *apo* sample is partially aggregated. (**c**) CPM unfolding experiment. The CCX777-extracted sample has a single, sharp transition, which is lacking in the *apo* sample.

A.

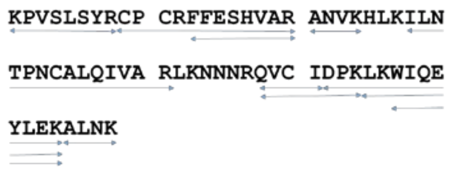

B.

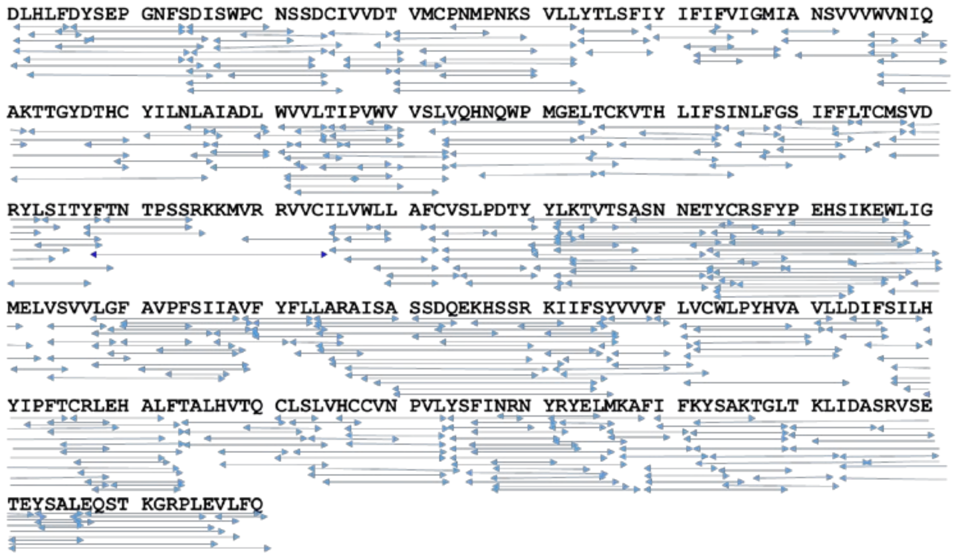

C.

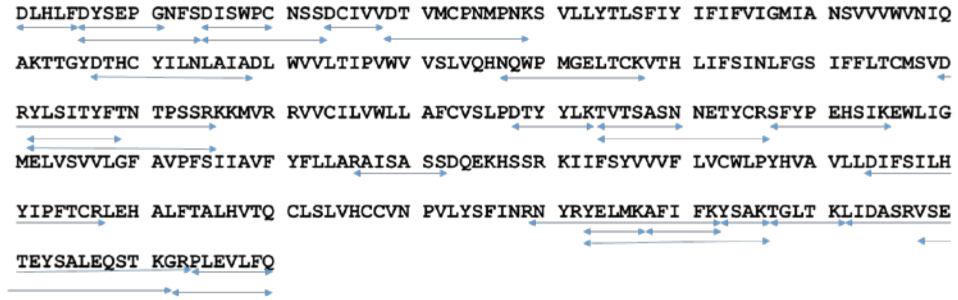

Supplementary Figure 2. (**a**) Sequence coverage of CXCL12 digested with Trypsin and AspN (87%). (**b**) Sequence coverage of ACKR3 digested with pepsin (100%). (**c**) Sequence coverage of ACKR3 digested with Trypsin and AspN (51%). Detected peptides are shown as blue arrows.

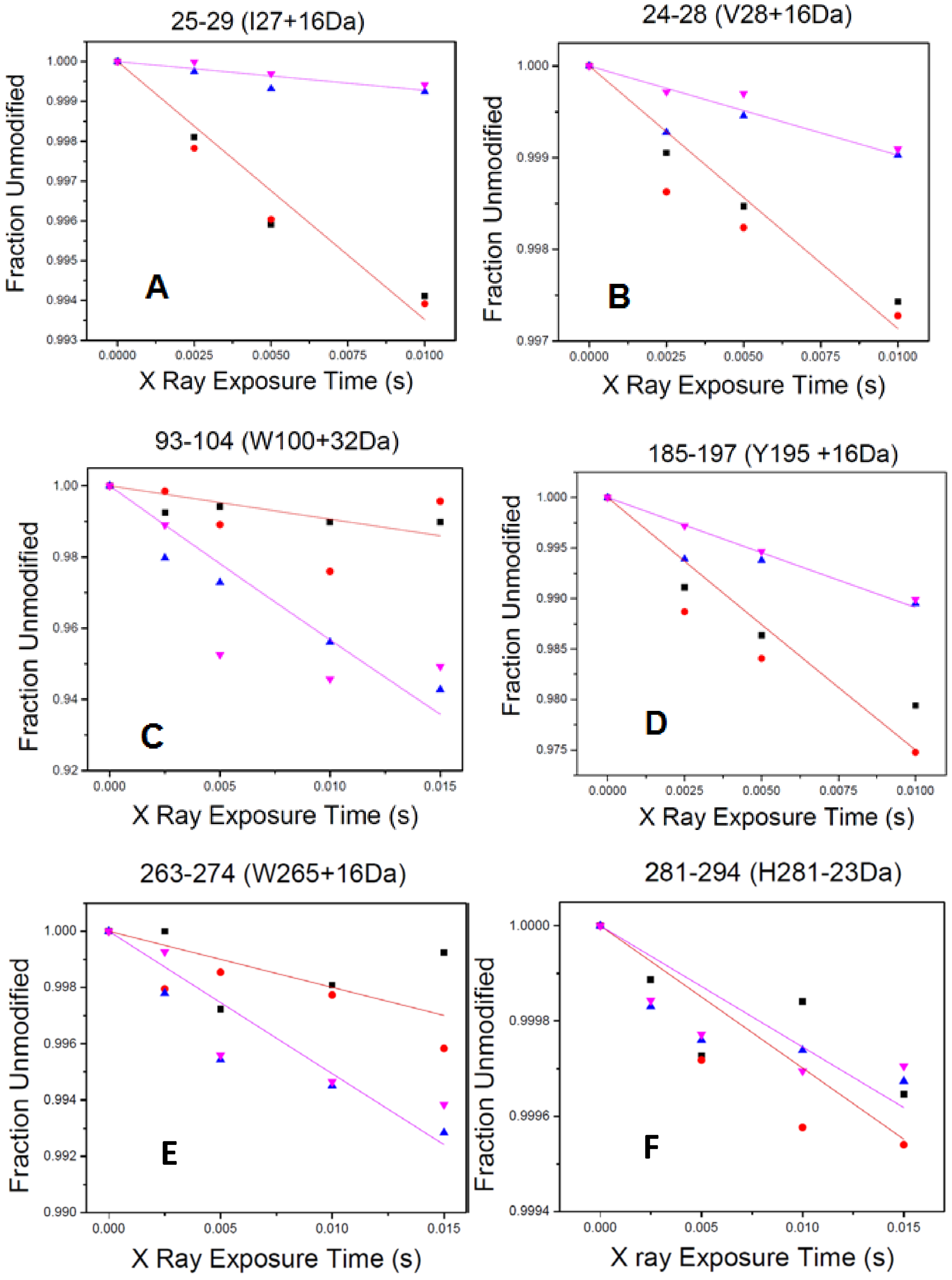
25-29 (I27+16Da)
Fraction Unmodified
1.000
0.999
0.998
0.997
0.996
0.995
0.994
0.993
A
0.0000
0.0025
0.0050
0.0075
0.0100
X Ray Exposure Time (s)
24-28 (V28+16Da)
Fraction Unmodified
1.000
0.999
0.998
0.997
B
0.0000
0.0025
0.0050
0.0075
0.0100
X Ray Exposure Time (s)
93-104 (W100+32Da)
Fraction Unmodified
1.00
0.98
0.96
0.94
0.92
C
0.000
0.005
0.010
0.015
X Ray Exposure Time (s)
185-197 (Y195 +16Da)
Fraction Unmodified
1.000
0.995
0.990
0.985
0.980
0.975
D
0.0000
0.0025
0.0050
0.0075
0.0100
X Ray Exposure Time (s)
263-274 (W265+16Da)
Fraction Unmodified
1.000
0.998
0.996
0.994
0.992
0.990
E
0.000
0.005
0.010
0.015
X Ray Exposure Time (s)
281-294 (H281-23Da)
Fraction Unmodified
1.0000
0.9998
0.9996
0.9994
F
0.000
0.005
0.010
0.015
X ray Exposure Time (s)

Supplementary Figure 3. Representative X-ray dose response curves for selected peptides/residues whose oxidation rates were decreased, increased or unchanged in the CXCL12-bound as compared to CCX777-bound ACKR3. Purple and blue triangles are replicates of ACKR3:CXCL12, red circles and black squares are replicates of ACKR3:CCX777. (**a**),(**b**) and (**d**) Oxidation rates of residues $I27^{N\text{-}term}$ and $V28^{N\text{-}term}$ in peptide 25-29 and $Y195^{ECL2}$ in peptide 185-197 from ACKR3:CXCL12 are lower than those from ACKR3:CCX777. (**c**),(**e**) Oxidation rates of residues $W100^{2.60}$ in peptide 93-104 and $W265^{6.48}$ in peptide 262-273 from ACKR3/CXCL12 are higher than those from ACKR3:CCX777. (**f**) Oxidation rate of $H280^{ECL3}$ in peptide 280-293 is similar for ACKR3:CXCL12 and ACKR3:CCX777.

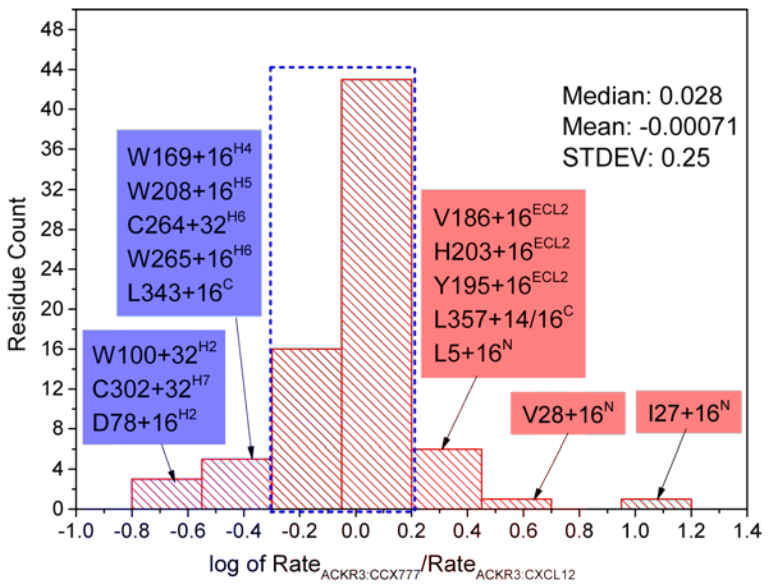

Supplementary Figure 4. Histogram of logarithms of oxidation rate ratios of ACKR3 residues between the ACKR3:CXCL12 and ACKR3:CCX777 complexes. 80% of the labeled residues exhibit values that are within the range of -0.3 to 0.2, highlighted by the dotted blue square located close to the median value (~0.03). Residues highlighted in blue (towards the left hand tail of the histogram) exhibit lower oxidation in ACKR3:CCX777 and include $W100^{2.60}$, $C302^{7.40}$, $D78^{2.38}$, $W169^{4.50}$, $W208^{5.34}$ $C264^{6.47}$, and $W265^{6.48}$ located in the TM domains and L343 in the C terminus. Residues highlighted in red include V186, H203, and Y195 from the receptor ECL2, L5, V28 and I27 from the N terminus, and L357 from the C terminus; they showed higher oxidation rates in ACKR3:CCX777 than in ACKR3:CXCL12.

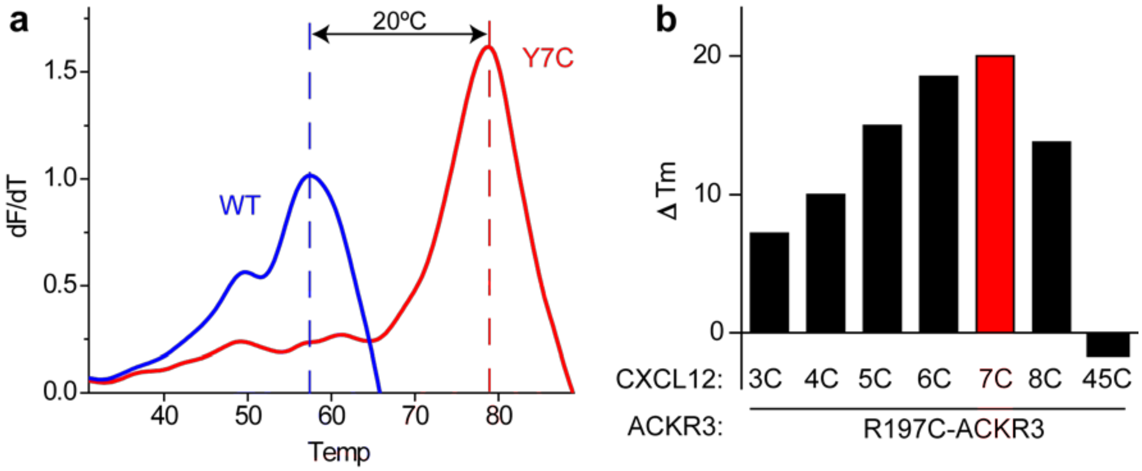

Supplementary Figure 5. Stability of disulfide-trapped complexes. (**a**) Derivative of CPM thermal unfolding curves for R197C-ACKR3:CXCL12 and R197C-ACKR3:Y7C-CXCL12 in DDM/CHS micelles. (**b**) Thermal stability of disulfide-trapped complexes. ΔTm was measured as $Tm_{\text{R197C-ACKR3:mutant-CXCL12}} - Tm_{\text{R197C-ACKR3:WT-CXCL12}}$.

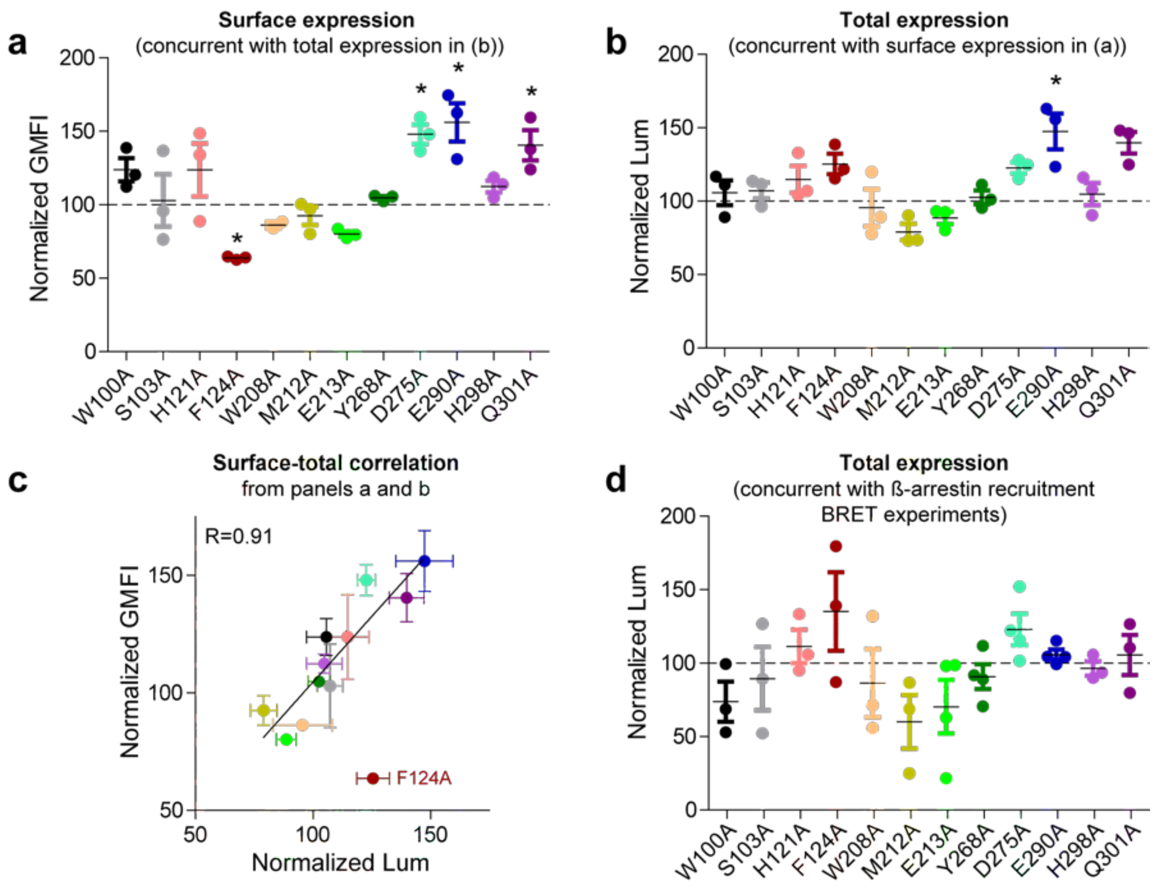

Supplementary Figure 6. Expression analysis of HA-ACKR3-Rluc3 mutants. (**a-b**) Surface and total expression of mutants were measured, in parallel, by flow cytometry following staining of the cells with allophycocyanin (APC) conjugated anti-HA antibody (a), and by luminometry (b), respectively. In (a), surface expression is shown as the geometric mean of the APC fluorescence intensity (GMFI) normalized to the corresponding measurement for HA-WT-ACKR3-Rluc3. In (b), total expression is shown as luminescence normalized to the luminescence of HA-WT-ACKR3-Rluc3. Each point corresponds to a biological replicate, the averages and standard errors of three biological replicates are shown as horizontal lines. Mutants with a mean expression that is significantly different from the wild type are marked by asterisks ($p < 0.05$ from one-way ANOVA with Dunnett's multiple comparison test). (**c**) Correlation between normalized surface and total expression for all HA-ACKR3-Rluc3 mutants from (**a**) and (**b**). Circles and error bars correspond to the average and standard error of three

biological replicates. Surface and total expression for all mutants excluding F124$^{3.32}$A are correlated with a correlation coefficient of R=0.91 as determined from linear regression. F124$^{3.32}$A has a low surface expression but WT-like total expression, suggesting that this mutant is partially internalized. (**d**) Total expression of HA-ACKR3-Rluc3 mutants in samples directly used for BRET experiments (see **Fig 6** and **Supplementary Table 5**) is shown as luminescence normalized to the luminescence of HA-WT-ACKR3-Rluc3. Each point corresponds to a biological replicate, average and standard errors of three biological replicates are shown as horizontal lines. No mutants have an expression that is significantly different from WT-ACKR3-Rluc3 as determined from paired t-tests.

As evidenced by this data, total expression levels were similar to WT for all mutants, and surface expression levels similar to WT for all mutants except F124$^{3.32}$A. The reduced surface expression of F124$^{3.32}$A (~60% of WT, **a** and **c**) is an obvious caveat in interpretation of the BRET β-arrestin-2 recruitment experiments for this mutant. Additionally, in the dedicated surface/total expression measurements (**a** and **b**), three mutants (D275$^{6.58}$A, E290^{ECL3}A and Q301$^{7.39}$A) have slightly higher total (although only E290A reached significance) and surface (all three significant) expression than WT ACKR3. In the case of E290^{ECL3}A and Q301$^{7.39}$A, this is irrelevant for BRET β-arrestin recruitment interpretation, as their total expression was not elevated in those experiments (**d**), and neither was their surface expression as suggested by the high surface/total expression correlation (**c**). In the case of D275$^{6.58}$A (which had a statistically non-significant ~20% increase in total expression in the BRET samples), lower E_{max} in the BRET β-arresting recruitment assay is only observed for CXCL12 but not for CCX777, indicating that this change in function is ligand-specific and not caused by the overall increased expression levels of the mutant. This analysis suggests that the observed changes in functional responses of mutants (possibly with the exception of F124$^{3.32}$A) to ligands are directly caused by

disruption of the inherent properties of the receptor (such as interaction with the ligands and/or activation capacity) and not by variations in receptor folding and trafficking.

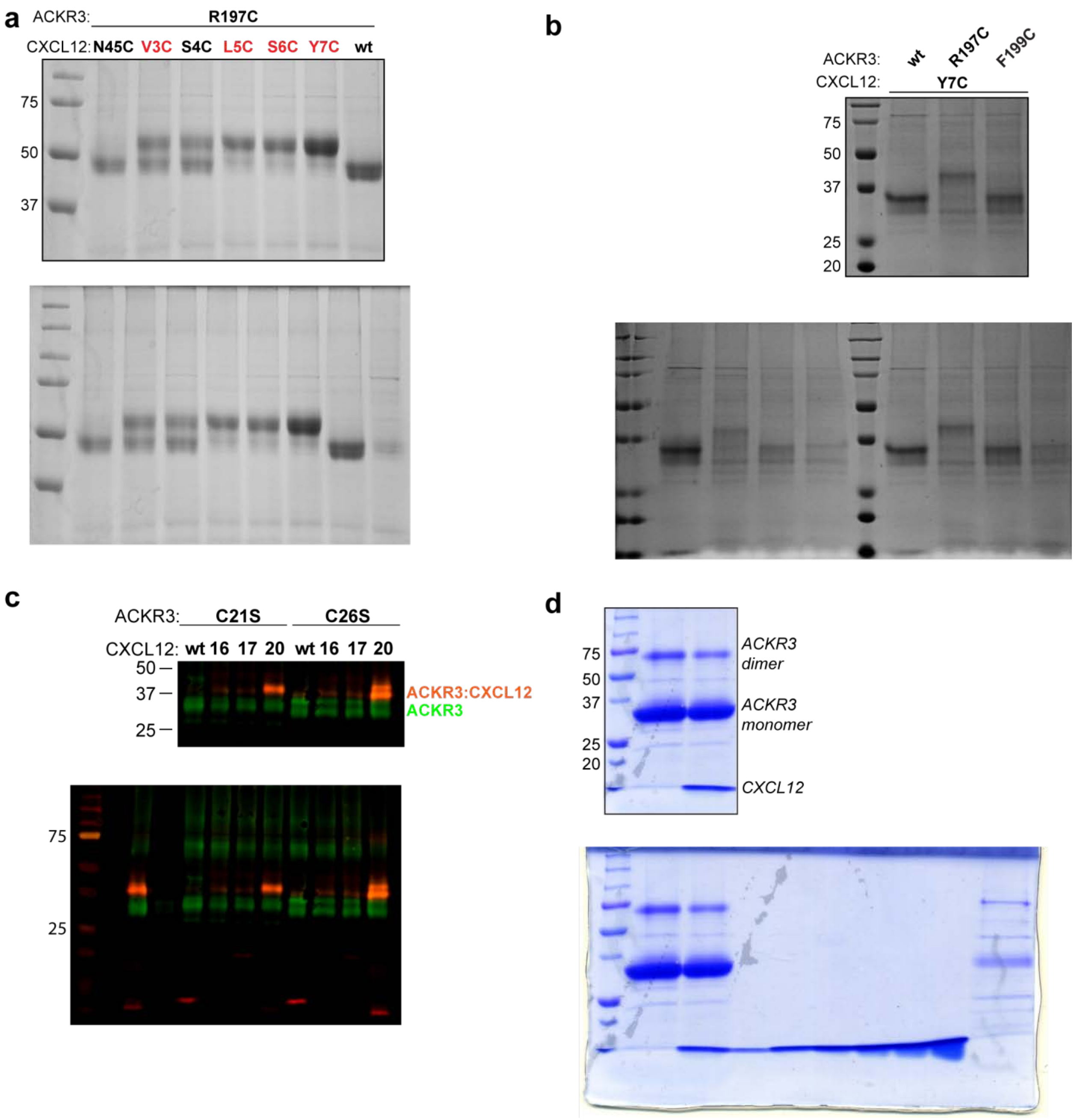

Supplementary Figure 7. Uncropped versions of gels and blots shown in main text. (**a**) Figure 5a, (**b**) Figure 5b, (**c**) Figure 5c, (**d**) Figure 2a.

Supplementary Table 1. Summary of oxidation rates measured for CXCL12 and comparison of rates for free CXCL12 and CXCL12 in complex with ACKR3

Peptide sequence	Residue range	Oxidized residue+mass shift (Da)	Oxidation rate k_{CXCL12}, s^{-1}	Oxidation rate $k_{ACKR3:CXCL12}$, s^{-1}	$k_{CXCL12}/k_{ACKR3:CXCL12}$
KPVSLSYR	1-8	L5+14	1.33±0.1	0.069±0.002	19.28
		L5+16	0.94±0.07	0.083±0.003	11.33
		Y7 &V3 +16	2.5±0.1	0.53±0.02	4.72
		K1/P2+16	0.6±0.03	0.029±0.004	20.69
FFESHVAR	13-20	R20-43+16	0.075±0.003	0.03±0.001	2.50
		H17-23	2.14±0.08	0.95±0.03	2.25
		V18+14	0.41±0.02	0.13±0.003	3.15
		F13/F14+16	1.88±0.1	0.53±0.03	3.55
		H17+16	4.1±0.4	2.56±0.1	1.60
ILNTPNCALQIVAR	28-41	R41-43+16	0.012±0.0006	0.0088±0.0004	1.36
		L36+14	0.28±0.03	0.17±0.01	1.65
		V39+14	0.26±0.01	0.062±0.006	4.19
		L29&P32+14	0.86±0.06	0.22±0.02	3.91
		Q37/I38+14	0.007±0.0003	0.00193±0.00002	3.63
		L36+16	0.49±0.04	0.28±0.014	1.75
		V39+16	0.065±0.004	0.011±0.0006	5.91
		L29+16	0.66±0.05	0.19±0.008	3.47
		P32+16	1.55±0.09	0.47±0.02	3.30
		I28+16	0.37±0.02	0.083±0.004	4.46
		C34+48	0.026±0.002	0.0034±0.0004	7.65
WIQEYLEK	57-64	E60-30	0.17±0.01	0.065±0.003	2.62
		E63-30	0.22±0.02	0.12±0.007	1.83
		L62+14	0.24±0.03	0.06±0.004	4.00
		E60+14	0.074±0.006	0.021±0.002	3.52
		E63/K64+14	0.12±0.01	0.058±0.004	2.07
		L62+16	0.85±0.1	0.43±0.01	1.98
		Y61+16	0.92±0.01	0.28±0.03	3.29
		W57+16	1.3±0.06	0.58±0.05	2.24
		E63/K64	1.1±0.02	0.48±0.04	2.29

Supplementary Table 2. Summary of ACKR3 radiolytic footprinting analysis including comparison of oxidation rates for ACKR3:CCX777 vs. ACKR3:CXCL12

Peptide sequence	Residue range	Oxidized residue+mass shift (Da)	Oxidation rate $k_{ACKR3:CCX777}$, s^{-1}	Oxidation rate $k_{ACKR3:CXCL12}$, s^{-1}	$k_{ACKR3CCX777}/k_{ACKR3:CXCL12}$
Pepsin digests					
FDYSEPGNF	6-14	Y8+16	0.17±0.02	0.11±0.07	1.55
		F6+16	1.4±0.1	1.4±0.05	1
		F14+16	0.76±0.03	0.66±0.02	1.15
		E10-30	0.036±0.003	0.045±0.004	0.8
YSEPGNFSD	8-16	F14+16	3.8±0.1	2.7±0.2	1.41
SDISWPCNSSD	15-25	W19+16	13.5±0.8	11±0.8	1.23
		W19+32	3.4±0.3	5.4±0.4	0.63
		C21+32	0.15±0.01	0.18±0.01	0.83
ISWPCNSSD	17-25	W19+16	2.1±0.1	2.7±0.3	0.78
		W19+32	1.2 ±0.04	0.90±0.05	1.33
		C21+32	0.18±0.005	0.22±0.01	0.82
CIVVD	26-30	V28+16	0.31 ±0.03	0.11±0.009	2.82
TVMCPNMPNKS	31-41	M37+16	3.8±0.3	7.9±0.6	0.48
		M33+16	34±0.3	36±4	0.94
WVVLTIPVW	92-100	W92+16	0.62±0.07	0.49±0.1	1.27
VVLTIPVWVVSL	93-104	W100+16	1.8±0.1	2.3±0.3	0.78
		W100+32	1.1±0.2	4.3±0.3	0.26
TIPVWVVSL	96-104	W100+16	2.2±0.6	3.6±0.7	0.61
		W100+32	1.3±0.1	4.4±0.3	0.3
VQHNQWPMGEL	105-115	M112+16	3.2±0.6	5.9±0.05	0.54
		W110+16	0.25±0.04	0.17±0.03	1.47
MSVDRYLSITY	138-148	M138+16	1.8±0.2	3.2±0.4	0.56
ILVWL	166-170	W169+16	1.3±0.4	4.4±0.3	0.3
CVSLPDTY	174-182	C174+32	0.72±0.5	0.82±0.06	0.88
YLKTVTSASNNET	182-194	Y182+16	N/A	N/A	
LKTVTSASNNETY	183-195	L183+16	N/A	N/A	
LKTVTSASNNET	183-194	V186/T187+16	0.045±0.005	0.025±0.004	1.8
YCRSFYPEHSIKE	195-207	H203+16	0.34±0.01	0.15±0.01	2.27
CRSFYPEHSIKE	196-207	F199+16	0.25±0.04	0.18±0.02	1.39
		H203+16	0.20±0.02	0.11±0.02	1.82
YCRSFYPEHSIKEWLIG	195-211	W208+16	≤3	9.1±1	≤ 0.33
WLIGME	208-213	M212+16	15±0.7	17±2	0.88
FAVPFSII	221-228	P224+16	0.013±0.002	N/A	
VCWLPYHVAVLL	263-274	C264+32	0.56±0.08	1.3±0.08	0.43
		W265+16	0.20±0.04	0.51±0.04	0.39
SILHYIPFTCRLEHALF	278-293	C287+32	3.5±0.4	2.8±0.1	1.25
HYIPFTCRLEHALF	281-294	P284/F285+16	0.22±0.02	0.18±0.04	1.22
		H281-23	0.025±0.03	0.030±0.04	0.83

HVTQCLSL	298-305	C302+32	0.67±0.08	2.9±0.4	0.23
		H298+16	< 0.02	N/A	
VHCCVNPVL	306-314	C308+32	0.072±0.01	0.11±0.009	0.65
		C309+32	0.20±0.02	0.37±0.05	0.54
		H307-22	N/A	N/A	
YSFINRNYRYEL	315-326	R320-43	0.14±0.007	0.14±0.009	1
		Y322+16	0.82±0.1	0.65±0.09	1.26
YSFINRNYRYELM	315-327	M326+16	1.7±0.2	2.7±0.4	0.63
INRNYRYEL	318-326	L326+16	0.025±0.001	0.021±0.002	1.19
		Y322+16	0.35±0.06	0.40±0.05	0.88
		R320-43	0.072±0.004	0.059±0.003	1.22
IFKYSAKTGL	331-340	F332+16	0.49±0.1	0.60±0.03	0.82
TKLIDASRVSE	341-351	L343+16	0.5±0.04	1.4±0.07	0.36
IDASRVSETE	344-353	V349+16	0.096±0.005	0.094±0.005	1.02
		E350/E352+16	0.20±0.006	0.19±0.006	1.05
		R348+14	0.031±0.004	N/A	
		E353-30	0.056±0.005	0.051±0.004	1.1
		E351-30	0.032±0.003	0.021±0.001	1.52
IDASRVSETEY	344-354	V349&E351+14	0.20±0.006	0.22±0.008	0.91
		V349&E351+16	0.39±0.01	0.42±0.01	0.93
		T352+16	0.25±0.01	0.22±0.009	1.14
		Y354+16	0.27±0.02	0.33±0.01	0.82
EQSTKGRPLEVL	358-369	L366+14	0.093±0.006	0.17±0.02	0.55
		L366+16	0.73±0.05	0.87±0.05	0.84
		K362+16	0.57±0.03	0.66±0.02	0.86
VLFQ	368-371	F370+16	1.6±0.07	1.8±0.1	0.89
		L369+16	1.6±0.04	1.9±0.06	0.84
**Trypsin+AspN digest**					
DLHLF	2-6	H4-23	0.20±0.009	0.20±0.008	1
		L5+16	0.38±0.03	0.21±0.007	1.81
		F6+16	1.1±0.02	0.91±0.01	1.21
		L3+16	0.37±0.02	0.25±0.007	1.48
		H4+16	0.50±0.02	0.58±0.03	0.86
DYSEPG	7-12	Y8+16	0.11±0.004	0.089±0.006	1.24
DYSEPGNFS	7-15	F14+16	2.5±0.2	2.1±0.1	1.19
DISWPC	16-21	I17+16	0.10±0.006	0.076±0.003	1.32
		W19+16	6.7±0.3	4.6±0.5	1.46
		W19+32	7.3±0.5	5.3±0.6	1.38
DCIVV	25-29	I27+16	0.65±0.03	0.072±0.009	9.03
		V28+16	0.29±0.02	0.10±0.01	2.9
		C26+32	1.0±0.03	0.85±0.09	1.18
DTVMCPNMPNK	30-40	M37+16	4.4±0.5	6.0±0.6	0.73
		M33+16	30±3.7	32±3.5	0.94
DTHCYILNLAIA	78-89	D78+16	0.27±0.05	1.2±0.08	0.23
YLSITYFTNTPSSR	143-156	I146+14	0.37±0.03	0.48±0.03	0.77

		P153+16	0.48±0.03	0.68±0.03	0.71
		I146&Y148+16	0.85±0.1	0.65±0.06	1.31
		R156-43	0.0061±0.0004	0.0097±0.0007	0.63
TVTSASNNETYCR	185-197	Y195+16	2.5±0.02	1.1±0.08	2.27
		N191+16	0.65±0.05	0.75±0.03	0.87
SFYPEHSIK	198-206	H203+16	0.34±0.02	N/A	
YELMK	324-328	M327+16	2.2±0.2	2.1±0.2	1.05
AFIFK	329-333	F332+16	0.56±0.02	0.59±0.007	0.95
VSETEYSALEQSTK	349-362	E358-30	0.10±0.005	0.094±0.008	1.06
		L357+14	0.72±0.04	0.36±0.01	2
		V349&K362+14	0.28±0.02	0.252±0.01	1.11
		L357+16	1.5±0.1	0.72±0.03	2.08
		Y354+16	1.7±0.07	1.2±0.08	1.42
		V349&K352	1.5±0.06	1.3±0.05	1.15
GRPLEVLFQ	363-371	R364-43	0.011±0.0006	0.0094±0.0009	1.17
		E367-30	0.14±0.008	0.11±0.006	1.27
		R364+16	0.0027±0.0002	0.0024±0.0002	1.13
		L369+14	1.3±0.04	0.91±0.04	1.43
		L365/V367+14	0.19±0.01	0.19±0.02	1
		L366+16	0.56±0.04	0.57±0.03	0.98
		F370+16	1.56±0.08	1.58±0.08	0.99
		L369+16	2.9±0.1	2.0±0.09	1.45

Supplementary Table 3. Protection factor analysis of CXCL12 sites for which +16 Da oxidation species were detected

	Free CXCL12				Bound CXCL12			
Oxidized residues (+16Da)	Oxidation rate, s^{-1}	ln PF	fSASA	fSASA (regressed)	Oxidation rate, s^{-1}	ln PF	fSASA (predicted)	ΔfSASA
K1+P2	0.60±0.03	1.67	1.05	0.60	0.029±0.004	4.70	-0.21	-0.81
V3+Y7	2.5±0.1	1.72	0.79	0.59	0.53±0.02	3.27	0.18	-0.41
L5	0.94±0.07	1.54	0.97	0.64	0.083±0.003	3.97	-0.01	-0.65
F13+F14	1.88±0.1	2.48	0.66	0.39	0.53±0.03	3.74	0.05	-0.34
H17	4.1±0.4	0.82	0.82	0.83	2.56±0.1	1.29	0.70	-0.13
I28	0.37±0.02	2.48	0.38	0.39	0.083±0.004	3.97	-0.01	-0.40
L29	0.66±0.05	1.90	0.26	0.54	0.19±0.008	3.14	0.21	-0.33
P32	1.55±0.09	-0.44	1.04	1.17	0.47±0.02	0.76	0.85	-0.32
L36	0.49±0.04	2.19	0.38	0.46	0.28±0.014	2.75	0.31	-0.15
V39	0.065±0.004	3.38	0.07	0.15	0.011±0.0006	5.15	-0.33	-0.47
W57	1.3±0.06	2.59	0.16	0.36	0.58±0.05	3.40	0.14	-0.22
Y61	0.92±.01	2.57	0.28	0.36	0.28±0.03	3.76	0.05	-0.32
L62	0.85±0.1	1.64	0.29	0.61	0.43±0.01	2.33	0.43	-0.18
E63+K64	1.1±0.02	0.72	0.81	0.86	0.48±0.04	1.55	0.63	-0.22

Supplementary Table 4. List of peptides that confirm the presence of a disulfide between C21 and C26 of ACKR3

Peptide sequence	Residue range	Protease	Total score
ISWPCNSSDCIVV	17-29	Pepsin	164
ISWPCNSSDCIVVD	17-30		
ISWPCNSSDCIVVDT	17-31		
SDISWPCNSSDCIVV	15-29		
SDISWPCNSSDCIVVD	15-30		
SDISWPCNSSDCIVVDT	15-31		
SSDCIVVD<br>DISWPC	23-30<br>16-21		
DYSEPGNFSDISWPC<br>NSSDCIVV	7-21<br>22-29	Trypsin/AspN	213
DISWPC<br>DCIVV	16-21<br>25-29		
DISWPCNSS<br>DCIVV	16-24<br>25-29		
DISWPCNSSDCIVV	16-29		
NSSDCIVV<br>DISWPC	22-29<br>16-21		

Scores were calculated from Massmatrix[68].

Supplementary Table 5. Summary of potency and efficacy of ACKR3 mutants in ACKR3-Rluc3/YFP10-β-arrestin-2 BRET experiments

		CXCL12		***CCX777***	
**Mutant**	**B.W**	**Δ*pEC*50 ± SEM (n)**	***E*$_{max}$ (% of WT) ± SEM (n)**	**Δ*pEC*50 ± SEM (n)**	***E*$_{max}$ (% of WT) ± SEM (n)**
W100A	2.60	-0.78 ± 0.12 (3)*	98 ± 1 (3)	-1.73 ± 0.15 (3)*	108 ± 14 (3)
S103A	2.63	0.05 ± 0.03 (3)	74 ± 6 (3)*	-0.2 ± 0.1 (3)	95 ± 6 (3)
H121A	3.29	-0.06 ± 0.17 (3)	93 ± 8 (3)	-0.09 ± 0.06 (3)	105 ± 9 (3)
F124A	3.32	-0.17 ± 0.16 (3)	63 ± 13 (3)*	0.2 ± 0.17 (3)	53 ± 8 (3)*
W208A	5.34	-0.61 ± 0.11 (3)*	72 ± 8 (3)*	-1.18 ± 0.26 (3)*	107 ± 9 (3)
M212A	5.38	-0.1 ± 0.1 (3)	104 ± 12 (3)	0.07 ± 0.07 (3)	114 ± 5 (3)
E213A	5.39	-0.05 ± 0.09 (4)	74 ± 2 (4)*	0.14 ± 0.21 (4)	98 ± 4 (4)
Y268A	6.51	0.22 ± 0.03 (4)	56 ± 8 (4)*	-0.09 ± 0.07 (4)	61 ± 9 (4)*
D275A	6.58	-0.08 ± 0.22 (4)	70 ± 5 (4)*	0.04 ± 0.11 (4)	93 ± 10 (4)
E290A	7.28	0.04 ± 0.1 (4)	91 ± 7 (4)	-0.04 ± 0.11 (4)	114 ± 11 (4)
H298A	7.36	0.08 ± 0.03 (3)	96 ± 15 (3)	-0.22 ± 0.06 (3)	98 ± 13 (3)
Q301A	7.39	0.23 ± 0.06 (3)	74 ± 10 (3)*	0.01 ± 0.05 (3)	69 ± 13 (3)*

Numbers report the average and standard errors of the mean for ΔpEC_{50} ($pEC_{50,mutant} - pEC_{50,WT}$) and normalized E_{max} ($E_{max,mutant}$ / $E_{max,WT}$ ×100) from 3-5 independent experiments. Asterisks indicate that the mutant is significantly different from WT-ACKR3 ($p \leq 0.05$) as determined from paired two-sided t-tests.

Supplementary Table 6. All primers used in the study

Primer	Sequence
ACKR3_AscI	gagggcgcgccggatctgcatctcttcgac
ACKR3_FscI	cccaggccggcctttggtgctctgctc
ACKR3_BamHI_forward	ccggatccgccgccgccaccatgtacccat
ACKR3_BamHI_reverse	ccggatcccctggtttggtgctctgctccaaggc
ACKR3_W100A_forward	ctcaccatcccagtcgcggtggtcagtctc
ACKR3_W100A_reverse	gagactgaccaccgcgactgggatggtgag
ACKR3_S103A_forward	ccagtctgggtggtcgctctcgtgcagcac
ACKR3_S103A_reverse	gtgctgcacgagagcgaccacccagactgg
ACKR3_H121A_forward	cgtgcaaagtcacagccctcatcttctccatc
ACKR3_H121A_reverse	gatggagaagatgagggctgtgactttgcacg
ACKR3_F124A_forward	gtcacacacctcatcgcctccatcaacctc
ACKR3_F124A_reverse	gaggttgatggaggcgatgaggtgtgtgac
ACKR3_W208A_forward	cacagcatcaaggaggcgctgatcggcatg
ACKR3_W208A_reverse	catgccgatcagcgcctccttgatgctgtg
ACKR3_W212A_forward	gagtggctgatcggcgcggagctggtctcc
ACKR3_W212A_reverse	ggagaccagctccgcgccgatcagccactc
ACKR3_E213A_forward	ctgatcggcatggcgctggtctccgttgtc
ACKR3_E213A_reverse	gacaacggagaccagcgccatgccgatcag
ACKR3_Y268A_forward	gtctgctggctgcccgcccacgtggcggtg
ACKR3_Y268A_reverse	caccgccacgtgggcgggcagccagcagac
ACKR3_D275A_forward	gcggtgctgctggccatcttctccatcctg
ACKR3_D275A_reverse	caggatggagaagatggccagcagcaccgc
ACKR3_E290A_forward	cacctgccggctggcgcacgccctcttcacg
ACKR3_E290A_reverse	cgtgaagagggcgtgcgccagccggcaggtg
ACKR3_H298A_forward	ctcttcacggccctggctgtcacacagtgc
ACKR3_H298A_reverse	gcactgtgtgacagccagggccgtgaagag
ACKR3_Q301A_forward	gccctgcatgtcacagcgtgcctgtcgctg
ACKR3_Q301A_reverse	cagcgacaggcacgctgtgacatgcagggc
ACKR3_R197C_forward	caacaatgagacctactgttgctccttctaccccgagcac
ACKR3_R197C_reverse	gtgctcggggtagaaggagcaacagtaggtctcattgttg
ACKR3_F199C_forward	gagacctactgtcggtcctgctaccccgagcacagcatc
ACKR3_F199C_reverse	gatgctgtgctcggggtagcaggaccgacagtaggtctc
ACKR3_C21S_forward	cggacatcagctggccaagcaacagcagcgac
ACKR3_C21S_reverse	gtcgctgctgttgcttggccagctgatgtccg
ACKR3_C26S_forward	gcaacagcagcgacagcatcgtggtggac
ACKR3_C26S_reverse	gtccaccacgatgctgtcgctgctgttgc
CXCL12_BamHI	atcgacggatccatgaacgccaaggtcgtggtcg
CXCL12_XhoI	cctggagaaagctttaaacaagtaatgactcgagagctta
CXCL12_V3C_forward	gcgacgggaagccctgcagcctgagctacagatgc
CXCL12_V3C_reverse	gcatctgtagctcaggctgcagggcttcccgtcgc
CXCL12_S4C_forward	gacgggaagcccgtctgcctgagctacagatgccc
CXCL12_S4C_reverse	gggcatctgtagctcaggcagacgggcttcccgtc
CXCL12_L5C_forward	gacgggaagcccgtcagctgcagctacagatgccc
CXCL12_L5C_reverse	gggcatctgtagctgcagctgacgggcttcccgtc
CXCL12_S6C_forward	gcccgtcagcctgtgctacagatgccc
CXCL12_S6C_reverse	gggcatctgtagcacaggctgacgggc
CXCL12_Y7C_forward	cgtcagcctgagctgcagatgcccatgcc
CXCL12_Y7C_reverse	ggcatgggcatctgcagctcaggctgacg
CXCL12_S16C_forward	ctggcaacatggcattcgaagaatcggcatggg
CXCL12_S16C_reverse	cccatgccgattcttcgaatgccatgttgccag
CXCL12_H17C_forward	gccgattcttcgaaagctgtgttgccagagccaacg
CXCL12_H17C_reverse	cgttggctctggcaacacagctttcgaagaatcggc
CXCL12_R20C_forward	cgaaagccatgttgcctgcgccaacgtcaagcatc

CXCL12_R20C_reverse	gatgcttgacgttggcgcaggcaacatggctttcg
CXCL12_N45C_forward	gcccggctgaagaactgcaaca
CXCL12_N45C_reverse	gtcaatgcacacttgtctgttgc
